# HIV-PrivFed: Privacy–Utility–Fairness-Aware Federated Learning for HIV Risk Stratification and Testing Prioritization Under Non-IID Clinical Heterogeneity

**DOI:** 10.3390/diagnostics16213590

**Published:** 2026-10-30

**Authors:** Md Wahidur Rahman, Haitham Y. Adarbah, Atena Pasha, Afzel Noore

**Affiliations:** 1 Department of Electrical Engineering and Computer Science, Texas A&M University–Kingsville (TAMUK), Kingsville, TX 78363, USA; md_wahidur.rahman@students.tamuk.edu (M.W.R.); afzel.noore@tamuk.edu (A.N.); 2 Department of Psychology and Sociology, Texas A&M University–Kingsville (TAMUK), Kingsville, TX 78363, USA; atena.pasha@tamuk.edu

**Keywords:** HIV screening, federated learning, differential privacy, non-IID clinical heterogeneity, privacy–utility tradeoff, fairness-aware learning, healthcare machine learning

## Abstract

**Background/Objectives:** HIV screening from routinely collected behavioral data could help to prioritize testing across clinics that cannot centralize sensitive records; however, the utility, privacy, and client-level fairness of federated models on real non-IID data are poorly characterized. **Methods:** Using Sialon-II, a cohort of men who have sex with men in 13 European cities (13 natural clients; horizontal federated learning), we benchmarked ten conventional classifiers and a centralized residual MLP, compared local-only, FedAvg, and FedProx baselines, and evaluated HIV-PrivFed, a differentially private FedProx-based algorithm with client-by-client Rényi accounting and client-level fairness auditing (fairness is audited, not enforced); secure aggregation overhead was benchmarked in simulation. **Results:** The best conventional model reached an AUROC of 0.696 (AUPRC 0.235), statistically indistinguishable from logistic regression on the original partition. Federated learning exceeded local-only learning (AUROC 0.607 versus 0.565). Achieved worst-client privacy budgets (ε = 22.1–224.1) were far looser than the nominal targets and give no meaningful guarantee; accuracy was retained at these budgets (AUROC 0.629–0.650) but fell to 0.553 and 0.524 at ε=8 and 4. Client-level estimates were highly uncertain. **Conclusions:** Useful accuracy and a meaningful privacy guarantee did not coexist in this small-cohort setting; larger cohorts and external validation are needed before deployment.

## 1. Introduction

Human immunodeficiency virus (HIV) is a viral infection that attacks the human immune system, particularly immune cells involved in protecting the body against infections and other diseases [1]. Despite major advances in prevention, diagnosis, and antiretroviral therapy, HIV remains a substantial global public health challenge [2]. According to the World Health Organization (WHO), approximately 41.0 million people were living with HIV worldwide at the end of 2025, while 1.2 million people acquired HIV and approximately 570,000 died from HIV-related causes during the same year [1]. Moreover, only 88% of people living with HIV were estimated to know their HIV status in 2025, indicating that an important diagnostic gap remains despite improvements in testing and treatment coverage [1]. Early identification is critical, as timely diagnosis provides an entry point to treatment, prevention services, and risk reduction interventions while helping to reduce onward transmission. These global figures aggregate many different epidemics and do not describe any single population. The data analyzed in this study come from one specific key population: men who have sex with men (MSM) recruited in 13 European cities in the Sialon-II study [3,4]. Within this population, the cohort comprises only men (4716 participants after exclusions, of whom 10.5% were HIV-positive); age- and sex-disaggregated WHO estimates for men who have sex with men in the 13 study cities were not available to us in a form that can be matched to this cohort, so no quantitative comparison with the global figures is attempted. The present work concerns HIV risk stratification in this population and setting, and its results should not be read as representative of the global population of people living with HIV.

Moreover, HIV risk is influenced by interacting demographic, behavioral, social, and clinical factors that may be difficult to characterize using simple rule-based screening strategies. Machine learning (ML) provides a data-driven approach for identifying nonlinear relationships among such factors and estimating individualized HIV risk [5]. Recent studies have demonstrated the potential of ML for HIV screening and risk stratification across different populations. Kansiime et al. developed and validated HIV risk prediction tools across four African settings [6]. Alie and Negesse evaluated eight ML techniques using demographic and health information from 4502 adolescents in Ethiopia and demonstrated the feasibility of algorithm-assisted identification of individuals requiring targeted HIV testing [7]. Similarly, Latt et al. developed an artificial intelligence-based HIV/STI risk assessment tool and reported that the selected HIV risk threshold identified 86% of HIV cases with 86.0% sensitivity and 65.6% specificity [8]. More recently, Ji et al. investigated RF, gradient boosting, XGBoost, and ensemble models for HIV/STI risk prediction using public demographic and behavioral data [9]. Collectively, these studies demonstrate the potential of ML-based risk estimation to support targeted screening; however, such predictive tools should complement rather than replace laboratory-based HIV diagnosis.

Besides predictive accuracy, the use of HIV-related data raises substantial privacy, ethical, and social concerns. HIV status and associated behavioral information are highly sensitive and may expose individuals to stigma, discrimination, or other harms if confidentiality is compromised [10]. Garett et al. emphasized that artificial intelligence applications for HIV require careful consideration of privacy, trust, fairness, transparency, and the potential amplification of existing social inequities [11]. Conventional centralized ML typically requires participant-level information from multiple institutions or populations to be transferred to a common repository. Although centralized training can simplify optimization, pooling sensitive HIV-related information increases the amount of identifiable or linkable data concentrated at a single location. Consequently, privacy-preserving collaborative learning is especially relevant when geographically separated institutions or populations are expected to contribute to a common predictive model.

Federated learning (FL) provides one possible solution by enabling multiple clients to train a shared model while retaining their raw data locally. Instead of transferring participant-level records, participating clients perform local model optimization and transmit model updates for global aggregation. Nguyen et al. recently demonstrated the feasibility of lightweight FL for STI/HIV risk prediction using demographic and behavioral information and reported promising predictive performance while reducing the need to centralize sensitive records [12]. Building on this feasibility demonstration, the distinguishing contribution of the present work is in the joint evaluation of patient-level differential privacy, naturally occurring city-level heterogeneity, and cross-client performance disparities within a single federated HIV screening pipeline. Nevertheless, the broader healthcare-FL literature shows that substantial challenges remain before such systems can be considered mature. Teo et al. reviewed 612 healthcare FL studies and found that only 5.2% involved real-life applications, indicating a considerable gap between methodological development and practical deployment [13]. Moreover, Zhang et al. identified class imbalance, missing data, distribution shifts, non-standardized variables, and data heterogeneity as recurring methodological challenges in healthcare FL [14]. These issues are particularly important for HIV screening because disease prevalence, behavioral characteristics, cohort size, and risk factor distributions can differ substantially among participating populations.

However, keeping raw records locally does not necessarily provide a formal privacy guarantee. Model parameters and gradients exchanged during FL may still reveal information about the underlying training data, and additional privacy mechanisms are required for sensitive healthcare applications [15]. Differential privacy (DP) addresses this limitation by bounding the contribution of individual training records through gradient clipping and controlled randomization, while secure aggregation can limit direct inspection of individual client updates. On the other hand, stronger privacy protection can reduce predictive utility, creating a privacy–utility tradeoff that becomes particularly important when individual clients contain relatively small or imbalanced clinical cohorts. Pati et al. highlighted these privacy risks and the associated costs of mitigation mechanisms in healthcare FL [15]. A privacy-aware HIV screening system should report the achieved privacy budget together with predictive performance, and should not assume that data localization alone guarantees confidentiality.

Furthermore, non-identically and independently distributed (non-IID) client data introduce another important challenge. In real multi-site healthcare environments, clients may differ substantially in sample size, class prevalence, demographic composition, and risk-factor distributions. A global model can achieve acceptable aggregate performance while providing substantially weaker performance for individual clients or underrepresented populations. Zhang et al. demonstrated that fairness must be explicitly considered in federated digital health systems, as conventional FL may produce unequal performance across subpopulations [16]. Similarly, Wang et al. showed that personalization and client heterogeneity can directly affect fairness in healthcare FL [17]. Consequently, evaluation based only on global accuracy or AUROC may conceal clinically important disparities. HIV screening requires particular attention to minority-class sensitivity, false negative behavior, calibration, and cross-client performance, as an apparently strong global model may still underperform in specific geographic populations.

Three questions remain open for federated HIV screening on small, imbalanced, and heterogeneous clinical cohorts. (RQ1) How much predictive utility survives when record-level differential privacy is enforced? (RQ2) What privacy budget is actually achieved, and for which client, when accounting is performed for every client rather than being assumed from a noise multiplier? (RQ3) How do decentralization and privacy redistribute errors across clients? Related work on these questions is reviewed in Section 2.

Motivated by these limitations, this study developed HIV-PrivFed, a privacy–utility–fairness-aware federated learning framework for HIV screening under natural non-IID heterogeneity, in which “fairness-aware” denotes fairness-aware evaluation rather than a fairness term in the training objective (Section 3.4.9). The evaluation used the Sialon-II bio-behavioral study, which recruited participants across 13 European cities and provided linked demographic, behavioral, and laboratory-derived HIV information suitable for geographically distributed analysis [4]. Rather than creating artificial random clients from a centralized cohort, the participating cities were retained as natural federated clients to preserve cross-site differences in sample size and HIV prevalence. The study first establishes a comprehensive conventional ML benchmark using both augmented and real-only training strategies, followed by a lightweight residual multilayer perceptron and local-only, FedAvg, and FedProx configurations. HIV-PrivFed subsequently integrates patient-level differential privacy with federated optimization while quantifying the obtained privacy budget, communication overhead, and computational cost; secure aggregation and authenticated encryption are benchmarked as optional system-layer protections.

Finally, unlike studies that evaluate privacy, predictive utility, or fairness independently, this work examines these dimensions jointly. The evaluation emphasizes AUPRC, MCC, sensitivity, specificity, calibration, and client-level performance in addition to conventional accuracy and AUROC. It further measures the privacy–utility tradeoff across differential-privacy configurations, examines performance disparities across the 13 naturally heterogeneous clients, and evaluates secure update and inference overhead. This design addresses an important gap between promising HIV risk prediction models and the requirements of privacy-sensitive and geographically distributed screening environments. The main contributions of this study are as follows:

- A reproducible end-to-end comparison under one fully specified protocol of ten conventional classifiers, a centralized residual MLP, local-only, FedAvg, and FedProx learning, and HIV-PrivFed on 13 natural clients, with independent repetitions for the federated and neural arms and a genuinely held-out patient-level test set under a strict no-leakage protocol.
- HIV-PrivFed (DP-FedProx), an explicitly defined algorithm (residual MLP, focal loss, FedProx, and DP-SGD) that is separated from the baselines and from the optional system-layer protections, and whose proximal term is applied after privatization, a detail that otherwise silently disables FedProx under Opacus.
- Client-by-client privacy accounting with persistent Rényi-DP accountants, which reports worst-client budgets between 22.1 and 224.1 and shows that the smallest client determines the guarantee, together with the sensitivity of the budget to cohort size, δ, local epochs, and rounds, and an empirical membership inference audit.
- Controlled ablations that separate the effects of clipping, noise, optimizer, proximal weight, model class, augmentation, and number of clients from those of natural heterogeneity.
- A client-level fairness audit with 95% confidence intervals, an exploration of city-specific decision thresholds, and a testing prioritization analysis. Fairness is audited, not optimized.
- Corrected evaluation practice and a characterization of where the current privacy–utility frontier sits for small-cohort federated clinical data: out-of-fold decision thresholds for conventional models, thresholds that never use the test set, and secure aggregation and AES-GCM overhead benchmarked as a simulation.

The remainder of this paper is structured as follows: Section 2 reviews related work; Section 3 introduces the Sialon-II dataset, outlines the experimental design, and details the proposed HIV-PrivFed framework, including its differential privacy, secure aggregation, and fairness components; Section 4 reports predictive performance, privacy–utility tradeoffs, and cross-client fairness metrics across all evaluated configurations; Section 5 interprets the results in the context of related work and discusses practical implications, limitations, and threats to validity; finally, Section 6 summarizes the key contributions and identifies avenues for future research.

## 2. Related Work

This section positions the present study against the literature on eight topics: HIV risk prediction, federated learning (FL) for HIV and healthcare, FL under non-IID data, privacy-preserving FL, differential privacy (DP), secure aggregation and encrypted updates, fairness and client-level evaluation, and the conventional classifiers used as baselines.

### 2.1. Machine Learning for HIV Risk Prediction and Testing Prioritization

Machine learning models have been used to estimate individual HIV risk from demographic, behavioral, and clinical variables and to prioritize who should be offered testing [5]. Reported work covers risk prediction tools developed across four African settings [6], algorithm-assisted identification of Ethiopian adolescents who need targeted testing [7], an artificial intelligence-based HIV/STI risk assessment tool [8], and tree-based and ensemble models built on public demographic and behavioral data for men who have sex with men [9]. These studies train a single model on pooled data, but do not address situations in which sensitive records cannot be pooled or the ethical concerns that arise when AI is applied to HIV-related information [11].

### 2.2. Federated Learning for HIV/STI Prediction and Healthcare

FL trains a shared model while the raw records remain at each participating site, and is usually realized by iteratively averaging locally trained models [18,19]. Lightweight FL has been shown to be feasible for STI/HIV risk prediction from demographic and behavioral information [12]. Systematic reviews of healthcare FL nevertheless report that few studies involve real-life deployment [13] and that class imbalance, missing data, distribution shift, non-standardized variables, and heterogeneity recur as methodological obstacles [14]. The present study does not repeat the feasibility demonstration of [12]. Its object of study is what happens to a federated HIV screening model when formal patient-level privacy, naturally occurring site heterogeneity, and client-level auditing are required at the same time.

### 2.3. Federated Learning Under Non-IID Healthcare Data

When client distributions differ, locally trained models drift apart and plain averaging can converge slowly or to a poor solution. FedProx addresses this by adding a proximal term that keeps each local model close to the current global model [20]. In healthcare, differences between clients also affect how performance is distributed across sites and subpopulations [17]. In Sialon-II, these differences are naturally present, as the 13 cities differ in size, HIV prevalence, and covariate distribution (Section 4.1).

### 2.4. Privacy-Preserving Federated Learning

Keeping data local does not necessarily represent a privacy guarantee, as shared updates can leak information about the training data and mitigation mechanisms carry costs in utility and computation [15]. Three different protections are often grouped together and must be kept apart: differential privacy bounds what any released output reveals about a single record; secure aggregation prevents the server from inspecting an individual client’s update; and authenticated encryption protects messages in transit. Only the first provides a record-level privacy guarantee (Section 3.4.8).

### 2.5. Differential Privacy in Federated Learning

Differential privacy [21,22] is enforced during neural network training by clipping per-example gradients and adding Gaussian noise (DP-SGD) [23]. Cumulative privacy loss is tracked with accountants such as Rényi DP [24,25] or numerical privacy loss–random variable (PRV) composition [26], implemented in the Opacus library [27]. Differentially private learning is more sample-hungry than non-private learning [28], which is why small clinical cohorts are a demanding setting. Many reports specify only the noise multiplier or a target ε; the privacy budget that is actually achieved depends on the sampling rate, the number of local steps, and the number of rounds of each client, and has to be accounted for client-by-client (Section 3.4.4).

### 2.6. Secure Aggregation and Encrypted Model Updates

Secure aggregation lets a server learn only the sum of client updates, for example through pairwise additive masks that cancel in the aggregate [29]. Authenticated encryption such as AES-GCM [30] protects the confidentiality and integrity of updates in transit. Neither mechanism limits what the final model reveals about a training record. In this paper, pairwise masking and AES-GCM are benchmarked for their computational and communication overhead; they are not presented as a deployed cryptographic protocol (Section 3.4.8).

### 2.7. Fairness and Client-Level Evaluation in Federated Healthcare

Fairness has to be considered explicitly in federated digital health, because a model with acceptable average performance can underperform for particular sites or subpopulations [16,17]. Fairness can be built into FL training objectives [31], or it can be audited after training with group metrics such as the equal-opportunity (true positive rate) gap [32]. DP itself may redistribute error unevenly across groups [33]. Our fairness component is of the second kind: it is an audit of per-client performance and does not enter the training objective (Section 3.4.9).

### 2.8. Conventional Classifiers Used as Baselines

The ten conventional classifiers (Section 3.5) are logistic regression, a radial basis function support vector machine [34], random forest [35], extremely randomized trees [36], histogram-based gradient boosting [37], XGBoost [38], LightGBM [39], CatBoost [40], balanced random forest [41], and EasyEnsemble [42]. They were run with scikit-learn [43] and imbalanced-learn [44].

### 2.9. Related Unsupervised Federated Methods

Federated multi-view clustering has been proposed for unsupervised analysis in settings where different views of the data are held by different clients [45,46]; a recent survey has reviewed this area [47]. These methods are not direct equivalents of the supervised classification problem studied here, because all Sialon-II clients share one feature space and one laboratory-confirmed outcome (horizontal FL). They would become relevant if the behavioral, clinical, and laboratory views of the same participants were held by different institutions, or if unsupervised discovery of risk phenotypes across sites were of interest (Section 5.9).

### 2.10. Positioning and Novelty of HIV-PrivFed

HIV-PrivFed does not introduce a new optimizer, and its building blocks (focal loss, FedProx, DP-SGD, Rényi accounting, secure aggregation, AES-GCM) are all existing techniques. Its contribution is the way they are combined and evaluated on a naturally partitioned HIV cohort: (i) a single fully-specified protocol in which every federated arm uses the same model family and data; (ii) client-by-client privacy accounting that shows which client determines the patient-level guarantee and how the achieved budget depends on cohort size, δ, local epochs, and rounds; (iii) controlled ablations that separate the effects of clipping, noise, optimizer, proximal term, model class, augmentation, and number of clients from those of natural heterogeneity; (iv) an empirical membership inference audit alongside the formal accounting; and (v) client-level fairness auditing with confidence intervals. We did not identify earlier work that reports all of these together for an HIV screening cohort; this statement reflects the literature we reviewed, and is not the result of a formal systematic review.

## 3. Materials and Methods

This section presents the overall architecture of the proposed solution, including the dataset findings, processing, and proposed HIV-PrivFed architecture. Figure 1 walks through the full HIV-PrivFed pipeline in four stages, moving from raw clinical data to a validated global model. Stage 1 starts with real non-IID data from the Sialon-II cohort, collected across K=13 European cities that serve as natural federated clients. Because each city differs in population size, HIV prevalence, and behavioral risk profile, the data are highly varied. Before training, records pass through preprocessing consisting of Winsorization, log transformation, robust scaling, and KNN imputation, then are split into HIV-positive and HIV-negative train/test sets [48]. Stage 2 is carried out independently for each client: a lightweight ResMLP-DP network (≈9857 parameters) is trained using DP-SGD, where per-example gradients are clipped, injected with Gaussian noise, and averaged before updating local weights. A focal loss and FedProx objective handles class imbalance and client drift, while an RDP accountant tracks cumulative privacy loss ε at $\delta =10^{-5}$. Stage 3 sends only these updates—never raw data—to an aggregation server, which combines them into a new global model via weighted FedAvg. Secure aggregation and encryption are optional system-layer protections, and their overhead is benchmarked in simulation (Section 3.4.8). Stage 4 evaluates the resulting global model on the disjoint patient-level held-out Sialon-II test partition, producing a confusion matrix (TP/FP/FN/TN) for performance evaluation. The bottom panel ties these stages to the framework’s broader contributions: the privacy guarantee, privacy–utility trade-off, fairness-aware evaluation across clients, system security, and scalable privacy-preserving HIV screening across Europe. Section 3.1 defines precisely which components are part of the proposed algorithm.

### 3.1. Study Design, Component Roles, and Definition of HIV-PrivFed

This study uses horizontal federated learning: all clients share the same 51-dimensional feature space and the same laboratory-confirmed outcome, but hold different participants. The methods are organized in three layers. Conventional classifiers, a centralized residual MLP, local-only learning, FedAvg, and FedProx are baselines; HIV-PrivFed is the proposed learning algorithm; secure aggregation and AES-GCM encryption are system-layer protections, and their overhead is benchmarked. The fairness analysis is an evaluation protocol. Table 1 assigns every component to exactly one role and states which security property it provides, if any.

**Definition 1.** 
*HIV-PrivFed (DP-FedProx) denotes the learning algorithm formed by a residual MLP with group normalization (ResMLP-DP), a class-weighted focal loss, FedProx proximal regularization, and DP-SGD (per-example gradient clipping, Gaussian noise, and Rényi-DP accounting) with sample-size-weighted aggregation. Secure aggregation and AES-GCM are optional transport-layer protections around this algorithm; they do not change its (ε,δ) guarantee and are not part of the training procedure. This definition is used consistently in all tables, algorithms, figures, and text. In ablations, “clipping-only” denotes the same algorithm with the noise multiplier set to zero, and “non-private FedProx” denotes it with neither clipping nor noise.*

### 3.2. Sialon-II MSM HIV Dataset

The experiments use the participant-level Sialon-II bio-behavioral HIV dataset, a multi-city survey of men who have sex with men (MSM) conducted during 2013–2014 across 13 European cities [3]. MSM constitute a key population for HIV surveillance in Europe and are difficult to reach through conventional population-based sampling; therefore, participants were recruited using venue-based time–location sampling and respondent-driven sampling within MSM community networks, which are methods specifically suited to this population. The original supplementary dataset contains 4901 participants recruited through this design. Each participant completed a self-administered behavioral questionnaire and provided a biological specimen for laboratory HIV antibody testing. The biological outcome was used as the reference label rather than self-reported HIV status. After excluding 185 records with invalid laboratory results, 4716 participants remained, comprising 497 HIV-positive (*Reactive*) and 4219 HIV-negative (*NotReactive*) observations.

Because the cohort is drawn exclusively from MSM recruited through community- and venue-based sampling rather than general population or clinical screening pathways, the models developed here reflect behavioral HIV risk stratification within this specific population and testing context. We restrict our claims accordingly: reported performance is most directly relevant to MSM and similar community-based testing settings, and should not be assumed to generalize to other key populations (e.g., heterosexual cohorts, people who inject drugs, transgender populations) or to routine antenatal/general population screening without independent external validation on those populations. This limitation is revisited in Section 5.

To preserve the intended pre-test screening setting, variables that directly or indirectly disclosed HIV status were removed before model development. These exclusions included self-reported or derived HIV-status variables, diagnosis-awareness variables, antiretroviral treatment indicators, viral load variables, and questionnaire items encoding prior HIV test results. Participant identifiers were retained only for audit purposes; the study city was retained only as the natural federated client identifier, and was not supplied to the classifier. The leakage-safe feature set represented demographic, psychosocial, sexual behavioral, and substance use characteristics, including age, education, migration status, sexual orientation outness, perceived social attitudes, recent partner type, number of sexual partners, number of condomless anal intercourse partners, sexual role, and substance use indicators. After preprocessing and encoding, the classifiers receive d=51 input columns: four numeric variables (age, number of partners, number of condomless anal intercourse partners, number of substances used) and 47 binary or one-hot columns, including missingness indicators.

A strict participant-level 50:50 holdout was performed before any augmentation. Joint stratification by study city and HIV label preserved geographic and class distributions while preventing participant overlap between partitions. This produced 2358 real participants for model development and 2358 real participants for the unseen evaluation set. The two partitions satisfy

(1) $$D_{\mathrm{train}}\cap D_{\mathrm{test}}=\emptyset ,$$

with equal cardinality

(2) $$\left|D_{\mathrm{train}}\right|=\left|D_{\mathrm{test}}\right|=2358.$$

The original training half contained 248 HIV-positive and 2110 HIV-negative participants, whereas the unseen test set contained 249 HIV-positive and 2109 HIV-negative participants. The test partition was never augmented, resampled, or used to estimate preprocessing parameters.

**Rationale for a large held-out partition.** The test partition is large because the outcome is rare and the evaluation is client-level. With 497 positives in total, the 50:50 split leaves 249 positives for evaluation and between 4 and 35 per city (Table 9). A conventional 80:20 split would have left about 99 positives overall, and on average only 7.6 per city, with 5 of the 13 cities holding fewer than five, which would make every per-client estimate and confidence interval uninformative. The price is a smaller training half, so that each federated client holds on average about 110 participants after undersampling (Section 3.3). We quantify the variability introduced by one particular split with repeated stratified resampling (Section 4.2), and do not rely on a single partition for the conclusions about the conventional models.

#### Data Flow and Training Populations

Three related but distinct training populations appear in this paper, and must not be conflated; Figure 2 shows how they are derived, and Table 2 states which population each experimental family uses.

### 3.3. Robust Preprocessing and Training-Only Data Augmentation

A leakage-safe preprocessing pipeline was applied using parameters estimated exclusively from the real training partition. All steps are performed after the 50:50 holdout and in the following order: (1) Winsorization, (2) logarithmic transformation of count variables, (3) median–interquartile-range scaling, (4) *k*-nearest neighbor imputation, (5) missingness indicators and one-hot encoding, and (6) z-scoring of all 51 input columns. Winsorization limits the influence of extreme counts, the logarithm reduces skew while preserving order, robust scaling places variables on a common scale without being driven by outliers, imputation retains participants with partly missing answers, and the indicators preserve potentially informative non-response. First, heavy-tailed numerical variables were bounded using the 1st and 99th percentiles estimated from the training data [49]. For the *j*th numerical feature of the *i*th participant, Winsorization was defined as

(3) $$x_{ij}^{\left(w\right)}=\min\left[Q_{0.99,j},\max\left(Q_{0.01,j},x_{ij}\right)\right],$$

where $Q_{0.01,j}$ and $Q_{0.99,j}$ denote the corresponding lower and upper training set quantiles.

Count-valued variables describing sexual partners, condomless anal intercourse partners, and substance use were subsequently transformed using

(4) $$x_{ij}^{\left(\log\right)}=\log\left(1+x_{ij}^{\left(w\right)}\right),$$

which reduces the influence of highly skewed extreme observations while preserving their relative ordering.

The numerical features were then normalized using median–interquartile-range robust scaling [43]:

(5) $$z_{ij}=\frac{x_{ij}-\mathrm{median}\left(x_{j}\right)}{Q_{0.75,j}-Q_{0.25,j}+\xi }$$

where $Q_{0.25,j}$ and $Q_{0.75,j}$ represent the first and third quartiles, respectively, and ξ is a small constant used to prevent division by zero. All scaling statistics were estimated only from the training partition and subsequently applied unchanged to the unseen test data.

Missing numerical values were estimated using a seven-nearest-neighbor imputer with inverse distance weighting [48]. For a missing value of feature *j* in participant *i*, the imputed estimate was defined as

(6) $${\hat{z}}_{ij}=\frac{\sum_{k\in N_{7}\left(i\right)}a_{ik}z_{kj}}{\sum_{k\in N_{7}\left(i\right)}a_{ik}},$$

where

(7) $$a_{ik}=\frac{1}{d(i,k)+\xi }.$$

Here, $N_{7}\left(i\right)$ denotes the set of the seven nearest training neighbors of participant *i*, while d(i,k) denotes the corresponding feature-space distance. Binary missingness indicators were additionally retained so that potentially informative questionnaire non-response patterns were not eliminated during imputation. For categorical variables, missing and non-applicable responses were represented explicitly rather than being replaced with the modal category. A one-hot encoder fitted exclusively on the real training partition was then used to generate the final categorical representation. Finally, all 51 input columns were z-scored with the mean and standard deviation of the undersampled real training subset; the same transformation was applied to the augmented set, to every federated client, and to the test partition. In a deployed federation, these per-feature means and variances would have to be shared or computed by secure aggregation; in the present simulation they are pooled, which is a simplification.

Class balancing was restricted to the training partition. Because the dataset contains both numerical and categorical attributes and exhibits natural between-city heterogeneity, a city-conditioned Synthetic Minority Over-sampling Technique for Nominal and Continuous features (SMOTENC) [50] was employed. Synthetic HIV-positive observations were generated independently within each city, preventing interpolation between geographically distinct federated clients.

For the continuous feature space, SMOTENC follows the neighborhood interpolation principle:

(8) $$x_{\mathrm{syn}}=x_{i}+\lambda \left(x_{\mathrm{nn}}-x_{i}\right)$$

where

(9) λ∼U(0,1).

Here, $x_{i}$ represents an HIV-positive training observation and $x_{\mathrm{nn}}$ denotes one of its minority-class nearest neighbors. For categorical attributes, SMOTENC selects valid categorical states represented within the corresponding local minority neighborhood. City-specific augmentation targets were assigned proportionally to each city’s original HIV-positive distribution. This strategy preserved the natural geographic heterogeneity of the federated clients rather than artificially imposing identical local distributions.

To maintain equal training and evaluation cardinalities without contaminating the unseen holdout, the training partition was reconstructed to 2358 observations with an exact 1:1 class distribution. All 248 real HIV-positive training participants were retained and supplemented with 931 training-only SMOTENC observations. In parallel, 1179 real HIV-negative training participants were retained through proportional city-wise undersampling. Consequently, the final training dataset contains

(10) $$N_{\mathrm{train}}^{+}=N_{\mathrm{train}}^{-}=1179,$$

resulting in 2358 training observations.

Three related but distinct training cohorts are referenced across this manuscript, and they must not be conflated. The original real training partition (Section 3.2) comprises all 2358 real participants retained after the 50:50 holdout (248 positive, 2110 negative). The undersampled real subset consisting of 1427 real participants (248 positive, 1179 negative) who passed the city-proportional negative-class undersampling described above is reported per city in Table 9 under “Real”. The final augmented training set combines this undersampled real subset with 931 city-conditioned SMOTENC synthetic positive observations, yielding the balanced 1179/1179 set of Equation (10) above.

The average per-city cohort size in the undersampled real subset is approximately 110 participants, versus approximately 181 participants per city in the original real partition; the $n_{k}\approx 110$ approximation used in Section 3.4 refers specifically to the undersampled subset feeding the neural and federated models, not to an inherent property of the underlying Sialon-II data. The 931 discarded negative participants were not contained in the processed files available to us, so experiments on the full 2358-participant real partition could not be performed (Section 5.8). Because the privacy accounting depends only on the client sizes, we nevertheless computed the achieved budgets for the full-partition cohort sizes, taking the per-city test sizes as the training sizes (the split is stratified by city and label, so they are equal up to one participant; mean 181.4). At σ=0.8, the worst-client budget would be 76.6 instead of 92.9 (Table 19). In contrast, the unseen test dataset preserves the original real-world class distribution, containing 249 HIV-positive and 2109 HIV-negative participants. No synthetic sample was introduced into the test partition.

All generated samples were marked as such and excluded from performance evaluation. Automated validation further confirmed identical training and test schemas, zero participant overlap, zero synthetic observations in the unseen test set, and complete model input matrices after preprocessing. No generated HIV-positive sample was an exact duplicate of a real HIV-positive training observation. Moreover, the maximum standardized mean difference between the real and synthetic HIV-positive samples across continuous predictors was 0.036, indicating close marginal agreement while preserving a fully independent real-participant evaluation cohort.

#### Fidelity of the Synthetic Positives

Because only 248 real positives were used for seeding the 931 synthetic positives, we audited how well the synthetic samples reproduce the relationships among the variables (Table 3). Marginal distributions are close: the largest standardized mean difference among the four numeric variables is 0.036, no synthetic sample duplicates a real one, and the pairwise correlation structure of the 44 non-constant columns agrees well with that of the real positives (Pearson correlation between the two sets of pairwise correlations 0.87; mean absolute difference 0.079, compared with 0.097 between two random halves of the real positives). However, close agreement is not equivalence; a classifier can still distinguish synthetic from real positives (cross-validated AUROC 0.70 for logistic regression and 0.82 for a random forest). Synthetic samples lie closer to real positives than real positives lie to each other (median nearest neighbor distance 2.11 versus 4.95), which indicates lower dispersion. The proportions of binary columns differ by up to 19.7 percentage points (mean 5.3). Finally, the association between each feature and the HIV label is less similar to that in the independent real test set after augmentation (Spearman correlation 0.52) than in the real-only training data (0.79). Therefore, we regard the synthetic samples as approximate, use them only in explicitly labeled augmented arms, and never train or audit the differentially private models on them.

### 3.4. Proposed HIV-PrivFed Architecture

HIV-PrivFed couples a lightweight residual classifier with per-example differentially private optimization and proximal federated aggregation. This subsection specifies the network, client-level objective, differentially private local update rule, privacy accountant, and global aggregation step, followed by the complete pseudocode. The symbols used in the equations, in Algorithms 1 and 2, and in Figure 1 are listed in Table 4; each symbol has exactly one meaning.
**Algorithm 1** HIV-PrivFed (DP-FedProx): Server-Side Training**Require:** 
K=13 real non-IID clients with data ${\left\{D_{k}\right\}}_{k=1}^{K}$; rounds T=30; noise multiplier σ; clipping norm C=1.0; proximal weight μ=0.01; tuned learning rate $\eta =\eta^{*}$ (0.2 for the ResMLP, 0.5 for logistic regression; 0.005 in the as-submitted runs); local epochs E=3; $\delta =10^{-5}$**Ensure:** 
Global model $\theta^{\left(T\right)}$; per-client budgets $\varepsilon_{k}$; patient-level budget $\varepsilon =\max_{k}\varepsilon_{k}$ with selected order α1:Initialize global model $\theta^{\left(0\right)}$ (ResMLP-DP, Equations (11)–(15)); give each client an empty RDP accountant $A_{k}$2:**for** t=0,…,T−1 **do**3:    **for** each client k∈{1,…,K} **in parallel do**4:        $\left(\theta_{k}^{\left(t+1\right)},A_{k}\right)\leftarrow$ ClientUpdate $\left(k,\theta^{\left(t\right)},A_{k}\right)$5:    **end for**6:    $\theta^{\left(t+1\right)}\leftarrow \sum_{k=1}^{K}\frac{n_{k}}{N}\theta_{k}^{\left(t+1\right)}$                       ▷ Equation (24)7:**end for**8:$\varepsilon_{k}\leftarrow A_{k}.$ GetEpsilon (δ) for every *k*; $\varepsilon \leftarrow \max_{k}\varepsilon_{k}$              ▷ Equation (23)9:**return**$\theta^{\left(T\right)},\left\{\varepsilon_{k}\right\},\varepsilon$

#### 3.4.1. Network Architecture

Each client holds a copy of a residual multilayer perceptron, ResMLP-DP, mapping an input feature vector $x\in R^{d}$ (d=51 for Sialon-II) to a single logit. Batch normalization is unsuitable under per-example differential privacy because it mixes statistics across a batch; we use Group Normalization (GN), which normalizes each sample independently and is compatible with Opacus’s per-sample gradient computation [27,51,52]. The forward pass is:

(11) $$\begin{array}{cc} h_{1} & =\mathrm{ReLU}\left(\mathrm{GN}(W_{1}x+b_{1})\right),\;W_{1}\in R^{64\times d} \end{array}$$

(12) $$\begin{array}{cc} h_{2} & =h_{1}+\mathrm{ReLU}\left(\mathrm{GN}(W_{2}h_{1}+b_{2})\right),\;W_{2}\in R^{64\times 64} \end{array}$$

(13) $$\begin{array}{cc} h_{3} & =\mathrm{ReLU}\left(W_{3}h_{2}+b_{3}\right),\;W_{3}\in R^{32\times 64} \end{array}$$

(14) $$\begin{array}{cc} z & =W_{4}h_{3}+b_{4}\in R,\;W_{4}\in R^{1\times 32} \end{array}$$

(15) $$\begin{array}{cc} \hat{y} & =\mathrm{sig}\left(z\right)=\frac{1}{1+e^{-z}} \end{array}$$

Equation (12) is the residual block. It adds its own (normalized, activated) transformation back to its input rather than replacing it, which stabilizes gradients through the otherwise shallow client-side network [53,54]. The full model has approximately 9857 trainable parameters. This is deliberately kept small, since differentially private training is more sample-inefficient than standard training [28] and each client’s real local cohort is small ($n_{k}\approx 110$ participants in the undersampled real subset; Section 3.3). The same architecture is used by every federated arm (local-only, FedAvg, FedProx, clipping-only, and HIV-PrivFed) and by the centralized ResMLP, ensuring that any differences between them are not caused by the network. As a model-class control, a logistic regression (a single linear layer with 52 parameters) is trained under the same federated protocols.

#### 3.4.2. Client-Level Objective: Focal Loss with FedProx Regularization

Each city’s HIV prevalence is heavily imbalanced (Table 9). For client *k*, let $w_{k}=n_{k}^{-}/n_{k}^{+}$ be the ratio of negative to positive examples in that client’s real data. For a predicted probability $p_{i}=\mathrm{sig}\left(z_{i}\right)$ and label $y_{i}$, define

(16) $$p_{i}^{★}=\left\{\begin{array}{cc} p_{i} & \mathrm{if}\;y_{i}=1\\ 1-p_{i} & \mathrm{if}\;y_{i}=0 \end{array}\right.$$

and the class-weighted binary cross-entropy

(17) $$\ell_{i}^{\mathrm{BCE}}=-\left[w_{k}\;y_{i}\log p_{i}+\left(1-y_{i}\right)\log\left(1-p_{i}\right)\right].$$

The per-example task loss is the focal loss [55]-style reweighting of Equation (17), which downweights already well-classified examples and concentrates gradient signal on hard (typically minority-class) examples:

(18) $$\ell_{i}\left(\theta \right)={\left(1-p_{i}^{★}\right)}^{\gamma }\;\ell_{i}^{\mathrm{BCE}},\;\gamma =2.$$

To keep each client’s local model from drifting too far from the current global consensus under heterogeneous non-IID city data, we add the FedProx [20] proximal term, giving the full local objective solved at round *t* as follows:

(19) $$J_{k}\left(\theta \right)=\frac{1}{n_{k}}\sum_{i\in D_{k}}\ell_{i}\left(\theta \right)+\frac{\mu }{2}\;{∥\theta -\theta^{\left(t\right)}∥}_{2}^{2},\;\mu =0.01$$

where $\theta^{\left(t\right)}$ is the global model broadcast at the start of round *t* and $D_{k}$ contains only real (is_synthetic=0) records for client *k*. Lowercase $\ell_{i}$ always denotes the loss of one example and calligraphic $J_{k}$ the objective of one client.

#### 3.4.3. Differentially Private Local Update

Client *k* minimizes Equation (19) using DP-SGD [23] via Opacus [27], which enforces patient-level privacy by clipping and noising per-example gradients rather than the batch-averaged gradient. With batch size parameter $B_{k}=\min\left(32,n_{k}-1\right)$, each local step draws a lot S by Poisson sampling with rate $q_{k}=1/\left\lceil n_{k}/B_{k}\right\rceil$, and one local epoch consists of $\left\lceil n_{k}/B_{k}\right\rceil$ steps. For each example i∈S, let $g_{i}=\nabla_{\theta }\;\ell_{i}\left(\theta \right)$ be the gradient of the per-example task loss. Each is clipped to a fixed $\ell_{2}$ norm bound *C*:

(20) $${\bar{g}}_{i}=g_{i}\cdot \min\;\left(1,\;\frac{C}{{∥g_{i}∥}_{2}}\right),\;C=1.0$$

after which Gaussian noise calibrated to the clipping bound and a noise multiplier σ is added to the sum of the clipped gradients and the result is divided by the expected lot size ${\bar{b}}_{k}=q_{k}n_{k}$:

(21) $$\tilde{g}=\frac{1}{{\bar{b}}_{k}}\left(\sum_{i\in S}{\bar{g}}_{i}+N\left(0,\;\sigma^{2}C^{2}I\right)\right).$$

The proximal term of Equation (19) depends only on the current local parameters and on the already released global model, not on any training record. It has no sensitivity to a participant, costs no privacy, and is applied after privatization:

(22) $$\theta \leftarrow \theta -\eta \left(\tilde{g}+\mu \left(\theta -\theta^{\left(t\right)}\right)\right)$$

with SGD learning rate η and E=3 local epochs per round. The learning rate $\eta =5\times 10^{-3}$ was used in the first submission; all reported private results use the tuned value $\eta^{*}$ chosen on inner validation data ($\eta^{*}=0.2$ for the ResMLP and $\eta^{*}=0.5$ for logistic regression; Section 4.5), and results with $\eta =5\times 10^{-3}$ are shown only for comparison. This placement is a necessary implementation detail, as Opacus replaces the accumulated gradient by the privatized per-example sum; thus, a proximal term added to the loss would be silently discarded and the procedure would reduce to DP-FedAvg. We verified numerically that the proximal term is inactive when added to the loss and active when applied as in Equation (22). We sweep σ∈{2.0,1.2,0.8,0.5}, which correspond to nominal target values of ε≈1,2,4,8 under a conventional design rule. The privacy budgets actually achieved are computed as described below, and are substantially larger (Table 5).

#### 3.4.4. Privacy Accounting

Rather than reporting the nominal σ-implied target, we track privacy loss with Opacus’s Rényi Differential Privacy (RDP) accountant [24,25,27]. Each client owns one persistent accountant that is updated at every executed local step with the pair $\left(\sigma ,q_{k}\right)$, meaning that composition runs over all local steps and all communication rounds ($\lceil n_{k}/B_{k}\rceil \cdot E\cdot T$ steps). The accountant converts the accumulated RDP curve to (ε,δ)-DP using the conversion of Balle et al. [56]:

(23) $$\varepsilon \left(\delta \right)=\min_{\alpha \in \Lambda }\left[\varepsilon_{\mathrm{RDP}}\left(\alpha \right)+\log\frac{\alpha -1}{\alpha }-\frac{\log\delta +\log\alpha }{\alpha -1}\right],\;\delta =10^{-5}$$

where the Opacus default grid Λ={1.1,1.2,…,10.9}∪{12,13,…,63} is searched automatically and the minimizing order α is reported. Each participant belongs to exactly one client, and the mechanisms of different clients act on disjoint data. The guarantee for a participant is the ε of that participant’s own client, and the patient-level guarantee of the federation is the maximum over clients. With a common batch-size parameter, the client that determines this maximum is the smallest one, since its sampling rate $q_{k}$ is largest (Bucharest, $n_{k}=59$, $q_{k}=0.5$). We report the worst-client value, the range across clients, and the selected α in Table 5; a numerical privacy loss random variable (PRV) accountant [26] is shown for comparison because it gives tighter values than RDP. Section 4.5 justifies the choice of $\delta =10^{-5}$ and quantifies its influence.

*Scope of the guarantee.* The accountant covers the releases of clipped and noised gradients, and so the local and global models derived from them; however, it does not cover several quantities that also depend on participants’ records: the class weights $w_{k}$, which are computed from each client’s label counts; the aggregation weights $n_{k}$; the Youden threshold, which is computed on pooled training predictions; the pooled z-score statistics; and the selection of the learning rate on training-derived validation data. These quantities would need their own noise or secure computation, so the (ε,δ) statement of this paper is restricted to the gradient releases and should be read as nominal.

#### 3.4.5. Global Aggregation

After local training, the server aggregates all K=13 client models, all of which participate in every round (participation rate 100%), weighted by local real-cohort size $n_{k}$, as in FedAvg [18]:

(24) $$\theta^{\left(t+1\right)}=\sum_{k=1}^{K}\frac{n_{k}}{N}\;\theta_{k}^{\left(t+1\right)},\;N=\sum_{k=1}^{K}n_{k}.$$

#### 3.4.6. Decision Threshold

Because class imbalance makes the default 0.5 threshold unsuitable, the operating threshold is selected once on training data only, via Youden’s *J* statistic [57], and is never tuned on the unseen test set:

(25) $$\tau^{*}=\arg\max_{\tau }\left[\;\mathrm{TPR}(\tau )-\mathrm{FPR}(\tau )\;\right].$$

For the federated and centralized neural models, the statistic is evaluated on the pooled training predictions of the final model. For the conventional classifiers, it is evaluated on out-of-fold predictions from stratified five-fold cross-validation on the training set, as in-sample predictions of high-capacity ensembles are nearly perfect and give degenerate thresholds.

#### 3.4.7. Algorithms

Algorithm 1 summarizes the complete HIV-PrivFed training procedure and Algorithm 2 details the differentially private client-side subroutine invoked at each round. Both use the notation of Table 4 and the equations above.
**Algorithm 2** ClientUpdate: Differentially Private Local Training    1:**function** ClientUpdate($k,\theta^{\left(t\right)},A_{k}$)    2:      $\theta \leftarrow \theta^{\left(t\right)}$; $w_{k}\leftarrow n_{k}^{-}/n_{k}^{+}$; $B_{k}\leftarrow \min\left(32,n_{k}-1\right)$; $q_{k}\leftarrow 1/\left\lceil n_{k}/B_{k}\right\rceil$; ${\bar{b}}_{k}\leftarrow q_{k}n_{k}$    3:      **for** local epoch e=1,…,E **do**    4:            **for** step $s=1,\ldots ,\lceil n_{k}/B_{k}\rceil$ **do**    5:                  draw lot $S\subseteq D_{k}$ by Poisson sampling, each example with probability $q_{k}$    6:                  **for** each example i∈S **do**    7:                        $z_{i}\leftarrow f\left(x_{i};\theta \right)$                    ▷ Equations (11)–(14)    8:                        $\ell_{i}\leftarrow {\left(1-p_{i}^{★}\right)}^{\gamma }\;\ell_{i}^{\mathrm{BCE}}$                  ▷ Equations (16)–(18)    9:                        $g_{i}\leftarrow \nabla_{\theta }\;\ell_{i}$; ${\bar{g}}_{i}\leftarrow g_{i}\cdot \min\left(1,\;C/{∥g_{i}∥}_{2}\right)$      ▷ per-example; Equation (20)10:                  **end for**11:                  $\tilde{g}\leftarrow \frac{1}{{\bar{b}}_{k}}\left(\sum_{i\in S}{\bar{g}}_{i}+N\left(0,\sigma^{2}C^{2}I\right)\right)$               ▷ Equation (21)12:                  $\theta \leftarrow \theta -\eta \left(\tilde{g}+\mu \left(\theta -\theta^{\left(t\right)}\right)\right)$       ▷ Equation (22); proximal term added after privatization13:                  $A_{k}.Step\left(\sigma ,q_{k}\right)$                    ▷ one accounted step14:            **end for**15:      **end for**16:      **return** $\theta ,A_{k}$17:**end function**

#### 3.4.8. Threat Model and Security Assumptions

The three protections in the framework answer different threats and are analyzed separately (Table 6). The adversaries considered are: (A1) an honest-but-curious aggregation server that follows the protocol but inspects every update and every global model, (A2) a passive network eavesdropper between a client and the server, and (A3) an analyst who obtains the released global model and attempts to infer whether a given person was in a client’s training data. Differential privacy addresses A1 and A3 at the record level only nominally; its guarantee is restricted to the clipped and noised gradient releases, and is not meaningful at the privacy budgets that are actually achieved (Section 5.1). Secure aggregation, if deployed with a complete protocol, addresses A1 with respect to individual client updates. AES-GCM [30] addresses A2. We assume that each participant contributes one record to one client, that the clients’ data are not shared, and that the test partition plays no role in training.

The following are not covered: malicious or Byzantine clients, including poisoning and backdoor attacks [58], collusion between the server and clients, active attacks on the cryptographic protocol, gradient inversion attacks [59] on updates exposed without effective differential privacy, key management and client authentication, side channels, and client dropout. The pairwise masking and AES-GCM components were implemented as a single-process simulation to measure overhead; they contain no key agreement, no secret sharing for dropped clients, and no authentication, and should not be read as a deployable secure aggregation protocol. Membership inference [60,61] is evaluated empirically in Section 4.5 as a complement to the formal accounting, not a substitute.

#### 3.4.9. Scope of the Fairness Component

“Fairness-aware” in the title refers to fairness-aware evaluation; the training objective of every model in this study contains no fairness term, models are trained to minimize the class-weighted focal loss, and fairness is audited after training. For each client *k*, we report the sensitivity (true positive rate), specificity, false positive rate, AUROC, and AUPRC, which are computed on that client’s test participants using the global decision threshold. Disparity is summarized by the largest value minus the smallest across clients, that is, the equal opportunity gap [32] for sensitivity and the corresponding gaps for the false positive rate and AUROC. Clients with fewer than ten HIV-positive test participants are flagged as statistically unstable, and are excluded from the stable client gap. Fairness-constrained training objectives [31] are left for future work (Section 5.9).

### 3.5. Experimental Protocol, Implementation, and Statistical Analysis

#### 3.5.1. Experimental Families

Five families of experiments are reported: (i) ten conventional classifiers trained on the undersampled real subset (“real-only”) or on the augmented set (“augmented”), with a logistic-regression repeated-split check of split dependence; (ii) a centralized ResMLP trained with a class-weighted focal loss or a class-weighted binary cross-entropy on the same two populations; (iii) federated baselines on the 13 natural clients, consisting of local-only learning, FedAvg, and FedProx plus a non-private FedProx variant that uses the optimizer of the private runs; (iv) HIV-PrivFed and its controls, consisting of clipping-only (noise multiplier zero), a sweep over four noise multipliers, and a one-factor sensitivity analyses of the clipping norm, learning rate, local epochs, rounds, and proximal weight, together with logistic regression counterparts; and (v) controls for heterogeneity and augmentation, consisting of regrouping the clients into two, three, or five clients and into IID clients with the same total population, followed by federated training on the augmented set. Table 2 states the population used by each family.

#### 3.5.2. Conventional Classifiers

Every classifier was run with fixed a priori hyperparameters (Table 7); no hyperparameter search was performed, which is a limitation discussed in Section 5.8. Features were z-scored with statistics of the respective training set. Class imbalance was handled with class weights or balanced resampling inside the learner. Each final model was fitted once on the full training set. The decision threshold was chosen by Youden’s statistic on out-of-fold predictions from stratified five-fold cross-validation on the training set (stratified by label, not city); the test partition played no role in tuning, thresholding, or model selection. For the augmented arm, the out-of-fold thresholds are optimistic because synthetic neighbors of a held-out real participant remain in the training folds.

#### 3.5.3. Federated and Neural Training

Table 8 lists all hyperparameters. All 13 clients participate in every one of the 30 communication rounds. Non-private local training uses Adam with five local epochs, a learning rate of $10^{-3}$ of the first submission, and a learning rate chosen on the same inner-validation protocol as the private arms (SGD on the grid of Table 20, and Adam on a grid from $10^{-4}$ to $10^{-2}$; Table 17). Private and clipping-only runs use SGD with a learning rate of $5\times 10^{-3}$ and three local epochs, as required by the DP-SGD formulation. A non-private SGD arm with the private optimizer settings separates the effect of the optimizer from the effects of clipping and noise. Local-only models are trained for 200 epochs per client with Adam and weight decay $10^{-4}$, and their threshold is chosen on that client’s training predictions. The centralized ResMLP uses Adam, weight decay $10^{-4}$, a batch size of 128, and 200 epochs. Network weights use the PyTorch default initialization; the global model is initialized from the run seed. No early stopping or checkpoint selection is used; the model after the last round is the reported model. Per-round test AUROC and AUPRC are logged only to draw convergence curves, and are never used for selection.

#### 3.5.4. Implementation and Reproducibility

All experiments were run in one environment: Python 3.12.3 on Linux, PyTorch 2.14.1 [62], Opacus 1.6.0 [27], scikit-learn 1.8.0 [43], imbalanced-learn 0.14.2 [44], XGBoost 3.4.1, LightGBM 4.7.0, CatBoost 1.2.10, PyCryptodome 3.24.1, NumPy 2.4.4, pandas 3.0.2, and SciPy 1.17.1, on one virtual CPU core (Intel Xeon at 2.80 GHz) with 4 GB of RAM and no GPU, under Ubuntu Linux 24.04. Federated and neural results are means and standard deviations over five independent runs (seeds 42–46) for the main comparison and over three runs (seeds 42–44) for the sensitivity and client count analyses; each seed controls model initialization, mini-batch order, Poisson sampling, and noise. The seed also controls the random assignment of participants to IID clients and of cities to random groups. Conventional classifiers use a fixed seed of 42 because their sources of randomness are small relative to the test sample variability that is quantified with the bootstrap. The code and the results of every run are provided with this submission (see the Data Availability Statement). To check that the code reproduces the reported numbers, selected runs (the privacy budget mapping, main federated comparison, and client count analysis) were re-executed from a clean copy of the packaged code and compared with the stored results; the outcome is given in the Supplementary Materials together with a SHA-256 manifest of all code and result files.

#### 3.5.5. Evaluation Metrics

Because HIV-positive participants are the minority class, accuracy is reported only for completeness. The primary threshold-free metrics are the area under the receiver operating characteristic curve (AUROC) and the area under the precision–recall curve (AUPRC), the latter being more informative under imbalance [63]; the baseline AUPRC of a random classifier equals the test prevalence of 10.6%. Threshold-dependent metrics are the sensitivity, specificity, Matthews correlation coefficient (MCC) [64], balanced accuracy, and F1. Calibration is summarized by the Brier score and the expected calibration error with ten bins of equal width.

#### 3.5.6. Statistical Analysis

*Uncertainty of a single model.* Confidence intervals are percentile intervals from 1000 bootstrap resamples of the test participants, resampling HIV-positive and HIV-negative participants separately so that the number of positives is preserved [65]. *Comparisons:* Two models are compared with a paired bootstrap in which the same resampled test participants are used for both models; for neural models, the difference is averaged over the seeds inside every resample so that the interval reflects test sample variability of the seed-averaged difference. A comparison is called significant when the 95% interval excludes zero. Four comparisons were designated as primary before the final analysis, and carry the conclusions: FedProx versus local-only learning, clipping-only versus HIV-PrivFed at σ=0.8, clipping-only versus HIV-PrivFed at a worst-client budget of ε=8, and HIV-PrivFed at σ=0.8 versus validation-tuned non-private FedProx; these are reported with their raw bootstrap *p*-values. All other comparisons are exploratory; for these, the table also gives *p*-values adjusted by the Holm step-down procedure [66] over the whole set of exploratory comparisons. Bootstrap *p*-values are limited by the number of resamples, and are reported as p<0.001 when no resample crosses zero. As a second coarser view, we report the Wilcoxon signed-rank *p*-value over the paired seeds, which with five seeds cannot be smaller than 0.0625 and so cannot reach the 0.05 level. All comparisons are reported. *Client-level estimates:* Per-city sensitivity, specificity, AUROC, and AUPRC are accompanied by 95% percentile bootstrap intervals computed within each city; cities with fewer than ten positive test participants are flagged as unstable. *City-specific thresholds:* For an exploratory comparison with the single global threshold, a Youden threshold was obtained for each city from the global model’s predictions on that city’s own training participants. *Testing prioritization:* Participants are ranked by predicted risk; for the top fraction *q*, we report the share of all positives found, the positive predictive value, and the increase over the test prevalence.

*Empirical privacy audit.* A loss threshold membership inference attack [60,61] scores each participant by the negative per-example cross-entropy loss of the final global model. Members are the pooled real training participants, while non-members are an equal number of randomly drawn test participants. The attack AUROC is 0.5 when the attacker cannot distinguish the two groups. This is a weak attack, so an AUROC near 0.5 does not demonstrate privacy or replace the formal accounting.

*Heterogeneity.* Between-client shift is quantified for each city versus the rest of the federation with four measures: the Jensen–Shannon distance between the city’s label distribution and the pooled one [67], the mean absolute standardized mean difference over the 51 input columns, an unbiased maximum mean discrepancy with a Gaussian kernel and the median-distance bandwidth [68], and the cross-validated AUROC of a logistic regression classifier that separates the city from the rest [69]. A two-dimensional t-SNE embedding [70] is used for exploratory visualization only; it is not evidence of fairness or predictive performance.

*Split dependence.* To quantify the dependence of the conventional models on the single 50:50 partition, logistic regression and EasyEnsemble were evaluated with five repetitions of two-fold cross-validation, stratified by city and label, on the 3785 real participants available in the processed files (1427 training plus 2358 test participants). The pooled prevalence of this set (13.1%) differs from that of the test partition because the training half had its negatives undersampled; AUROC, sensitivity, and specificity are prevalence-invariant, whereas AUPRC is not.

## 4. Results

This section evaluates the proposed framework in terms of data heterogeneity, conventional machine learning performance, handling of class imbalance, federated learning, privacy–utility behavior, client-level variation, statistical robustness, and computational efficiency. All predictive evaluations use the held-out unseen test partition. Neural and federated results are means over independent runs with different random seeds (five for the main comparison and three for the sensitivity and client count analyses), and uncertainty is reported as the standard deviation over seeds or as a bootstrap interval over test participants, as stated for each table.

### 4.1. Dataset Distribution and Natural Non-IID Heterogeneity

Table 9 summarizes the augmented training set, the undersampled real participant component of that training set, and the unseen test distributions across the 13 cities in the Sialon-II dataset. Considerable variation is observed in both client size and HIV-positive prevalence. In the undersampled real training subset, HIV-positive prevalence ranges from 4.3% in Vilnius to 30.5% in Bucharest, while the unseen test prevalence ranges from 2.5% to 19.8%. These differences provide a naturally varied federated setting without artificially partitioning a centralized dataset.

The covariate shift between clients is also substantial (Table 10). A classifier that tries to separate one city from the rest of the federation using only the 51 covariates reaches a cross-validated AUROC between 0.71 (Ljubljana) and 0.92 (Sofia), with a mean of 0.80. The largest kernel discrepancies are found for Bucharest, Vilnius, and Sofia, and the label distribution of Vilnius deviates most from the pooled one (Jensen–Shannon distance 0.183). The exploratory t-SNE embedding in Figure 3 shows that participants from all cities occupy overlapping regions of the feature space while some cities concentrate in particular clusters. Because such an embedding is distorted by construction, we rely on the quantitative measures and do not use it as evidence of fairness or of predictive performance.

### 4.2. Conventional Machine Learning Performance

Table 11 reports the performance of the conventional machine learning models under augmented and real-only training, with the same model order in both blocks. The results demonstrate that overall accuracy alone is insufficient for evaluating HIV screening. Because the test prevalence is 10.6%, a classifier that labels every participant as HIV-negative reaches an accuracy of 0.894. In the augmented arm, tree-based and boosting models reach accuracy of 0.80–0.85 while detecting only 0.13–0.23 of the positive participants. In the real-only arm, the out-of-fold thresholds give balanced operating points (sensitivity 0.56–0.82, specificity 0.38–0.65) at accuracy of 0.43–0.64, all below that trivial benchmark. Accordingly, AUROC, AUPRC, MCC, balanced accuracy, and sensitivity are considered together.

Among the augmented-data experiments, EasyEnsemble achieves an AUROC of 0.6588 and an AUPRC of 0.1864. Under real-only training, EasyEnsemble reaches an AUROC of 0.6956 and an AUPRC of 0.2355, while logistic regression obtains an AUROC of 0.6919 and an AUPRC of 0.2134. The comparison indicates that training with synthetically balanced data does not consistently improve generalization to the real unseen population, with ten of the ten classifiers reaching a higher AUROC with real-only training than with augmentation.

Figure 4 compares the AUPRC values of the conventional models. The figure provides a more informative comparison than accuracy in the present imbalanced screening problem, as AUPRC directly reflects performance on the minority HIV-positive class. The observed variation also shows that a model with high overall accuracy does not necessarily provide the strongest positive-class discrimination.

Figure 5 presents the receiver operating characteristic and precision–recall curves of centralized EasyEnsemble and logistic regression under real-only and augmented training. The curves illustrate the tradeoff between true positive and false positive rates over the full range of classification thresholds; because HIV-positive participants constitute the minority class, the reduction in precision that occurs when sensitivity is increased. Federated models are compared with these references in Section 4.4.

As a multi-metric summary, Figure 6 profiles the four strongest real-only conventional models across six metrics that share a common 0–1 scale. The four models are close in AUROC (0.66–0.70) but differ in operating point: logistic regression and BRF favor sensitivity (0.747 and 0.755), whereas CatBoost (sensitivity 0.631, specificity 0.639) and EasyEnsemble (0.663 and 0.596) are more balanced.

To quantify how much these conclusions depend on the single 50:50 partition, Table 12 repeats the comparison of EasyEnsemble and logistic regression over ten stratified partitions. Across these partitions, EasyEnsemble reaches a mean AUROC of 0.696 (SD 0.014) and logistic regression of 0.664 (SD 0.012), and EasyEnsemble exceeds logistic regression in all ten partitions with a mean AUROC difference of +0.032. The original partition is one on which logistic regression performed unusually well (AUROC 0.6919 versus a repeated-split mean of 0.664), so the near-identical performance of the two models on that partition (Section 4.7) understates a modest but consistent advantage of EasyEnsemble. The ten partitions reuse the same participants, and are not independent; thus, we attach no significance test to this consistency.

### 4.3. Effect of Class Imbalance Handling and Augmentation

Table 13 evaluates the effect of focal loss, weighted binary cross-entropy, and the use of augmented or real-only training data on the centralized ResMLP (mean over five seeds), with three conventional models as labeled references. The real-only ResMLP with weighted BCE achieves an AUROC of 0.605 ± 0.013, whereas the corresponding augmented BCE configuration obtains 0.582 ± 0.014. With focal loss, the values are 0.601 ± 0.006 (real-only) and 0.577 ± 0.008 (augmented). These results indicate that the choice of class imbalance strategy changes the operating behavior of the classifier far less than the choice of training population does, and reinforce the observation that synthetic augmentation does not uniformly improve unseen-test generalization. Choosing the Adam learning rate of the centralized ResMLP on inner validation data (selected η=0.01, the edge of the grid) did not improve its test AUROC (0.591 ± 0.006 against 0.601 ± 0.006 with the original rate), so the centralized ResMLP is not better than the federated models of the same architecture in this study.

The same holds in the federated setting (Table 14). Training the 13 clients on city-wise augmented data instead of real-only data gives an AUROC of 0.598 ± 0.016 instead of 0.607 ± 0.009 for FedAvg and 0.597 ± 0.016 instead of 0.607 ± 0.011 for FedProx; the paired differences are −0.009 (95% CI (−0.027, +0.009)) and −0.011 (−0.027, +0.008). Together with the fidelity audit of Section Fidelity of the Synthetic Positives, which shows that the synthetic samples are distinguishable from real ones and weaken the feature–label associations, this answers the question of whether augmentation is needed in the negative for HIV-PrivFed. The private models are trained on real participants only, and we recommend the cost-sensitive real-only configuration.

### 4.4. Federated Learning Under Non-IID Heterogeneity

Table 15 compares local-only, FedAvg, FedProx, and the private variants under one protocol: the same GroupNorm ResMLP-DP and 13 real clients, 30 rounds, and the same decision rule. Local-only learning produces an AUROC of 0.565 ± 0.005 and an AUPRC of 0.132 ± 0.002. FedAvg increases these values to 0.607 ± 0.009 and 0.157 ± 0.006, respectively, while FedProx obtains an AUROC of 0.607 ± 0.011 and an AUPRC of 0.158 ± 0.007. The paired comparison in Table 16 gives a FedProx minus local-only AUROC difference of +0.042 with a 95% interval of (+0.017, +0.065), which excludes zero, so federated collaboration recovers useful cross-client information despite differing local data distributions. FedProx and FedAvg are indistinguishable (difference +0.000, interval (−0.001, +0.002)); with μ=0.01, the proximal term is too weak to change the result. These non-private arms used the learning rate of the first submission. When their learning rate is chosen on inner validation data (Table 17), FedAvg and FedProx reach an AUROC of 0.628 ± 0.007 (selected η=0.0003), which is higher than with the original rate (+0.020, interval (+0.008, +0.033)) and higher than local-only learning (+0.063, interval (+0.040, +0.085)); the comparisons with the private models below use these validation-tuned arms. The local-only models use thresholds fitted to their own heavily overfitted training predictions, so their sensitivity and specificity in Table 15 are not comparable with those of the federated models, and local-only and federated learning should be compared through AUROC and AUPRC.

Replacing the ResMLP by logistic regression (Table 18) isolates the effect of the model class. Under the same untuned Adam protocol, federated logistic regression reaches an AUROC of 0.647 ± 0.015, against 0.607 ± 0.011 for the federated ResMLP (paired difference +0.040, interval (+0.015, +0.064)), and also exceeds local-only logistic regression (0.560 ± 0.002). This gap shrinks when both models are trained with the tuned SGD protocol of Section 4.5 (differentially private logistic regression minus ResMLP at σ=0.8: +0.013, interval (−0.009, +0.036)), so the first gap reflects the optimizer settings of the ResMLP as much as the model class. Even so, the federated models (AUROC about 0.65–0.67 when tuned) remain below the centralized logistic regression (AUROC 0.6919), a difference that this study does not decompose further.

Figure 7 illustrates the round-wise behavior. The non-private FedAvg and FedProx curves peak at round 6 (AUROC 0.637) and then decline to 0.607 by round 30, which suggests overfitting to the small clients. Because the reported model is always the final-round model and no stopping rule was fitted on validation data, the non-private results in Table 15 may understate what early stopping would achieve; the round was deliberately not chosen from the test curve. Figure 8 shows the corresponding ROC and precision–recall curves of the federated models against centralized EasyEnsemble. Communication and computational requirements are summarized in Section 4.8.

### 4.5. Privacy–Utility Tradeoff

*Achieved privacy budgets.*Table 5 gives the mapping from noise multiplier to nominal target and to the achieved patient-level budget. At $\delta =10^{-5}$, the worst-client budgets are 22.1, 47.2, 92.9, and 224.1 for σ=2.0, 1.2, 0.8, and 0.5, with selected Rényi orders between 2.2 and 1.2; the best clients have budgets that are 20% to 34% lower. The nominal targets ε≈1–8 are not reached; Table 19 and Figure 9 show why. The budget falls steeply with the cohort size of the client (from 92.9 at $n_{k}=59$ to 20.4 at $n_{k}=1,000$ and 5.2 at $n_{k}=10,000$ for σ=0.8), grows with local epochs and rounds, and is only mildly sensitive to δ (98.7 at $\delta =10^{-6}$ against 81.1 at 1/N, a reduction of 12% to 18%). Reaching ε=8 for the worst client would require σ≈4.4, about 2.2 times the largest value in the original sweep. The choice $\delta =10^{-5}$ is about 70 times smaller than 1/N for the N=1,427 real training participants, as is conventionally required, and moving it from $10^{-6}$ to $10^{-4}$ or to 1/N lowers ε by only 12% and 18%, respectively.

*Learning rate and the cost of clipping and noise.* The learning rate turns out to be decisive; on inner validation data (Table 20), the validation AUROC of HIV-PrivFed at σ=0.8 rises from 0.538 at the learning rate of 0.005 used in the first submission to 0.623 at $\eta^{*}=0.2$. On the test set (Table 15), clipping-only training reaches an AUROC of 0.534 ± 0.036 at η=0.005 and 0.651 ± 0.010 at $\eta^{*}$, and the private runs at η=0.005 are indistinguishable from clipping-only (for σ=0.8: 0.534 ± 0.036 versus 0.534 ± 0.036; clipping-only minus private: +0.000, interval (−0.000, +0.001)). Without clipping, SGD with the same learning rate reaches 0.622 ± 0.010, so clipping does not reduce utility at the tuned learning rate and even raises it slightly (unclipped minus clipped: −0.028, interval (−0.047, −0.009)), whereas clipping lowers the AUROC at η=0.005 (unclipped minus clipped: +0.053). The near-chance private results of the first submission reflected an under-tuned optimizer, not privacy noise.

With the tuned learning rate, HIV-PrivFed reaches AUROC 0.629 ± 0.015, 0.647 ± 0.015, 0.650 ± 0.015, and 0.650 ± 0.014 at σ=2.0, 1.2, 0.8, and 0.5 (worst-client ε of 22.1–224.1), compared with 0.628 ± 0.007 for validation-tuned non-private FedProx (Adam) and 0.622 ± 0.012 for validation-tuned non-private SGD (η=0.1). Tuning the non-private arms removes about half of the advantage that the private model showed over the untuned baseline (HIV-PrivFed at σ=0.8 minus untuned FedProx: +0.043), but a significant residual remains (minus validation-tuned FedProx: +0.022, interval (+0.005, +0.039), p= 0.010; minus validation-tuned SGD: +0.028, interval (+0.009, +0.049)). This residual is not a benefit of privacy; the noise has no measurable effect for σ≤1.2, and clipping accounts for it (validation-tuned unclipped SGD minus clipping-only: −0.029, interval (−0.046, −0.010)). While we did not investigate why clipping helps, a regularizing or normalizing effect on the per-example updates is plausible. The difference between clipping-only and private training (clipping-only minus private) is +0.001, (interval −0.007, +0.008) at σ=0.8 and +0.000 (interval −0.005, +0.005) at σ=0.5. The private models are not worse than local-only learning: at σ=0.8, HIV-PrivFed exceeds local-only by +0.085 (interval +0.065, +0.106). The noise multipliers 1.2, 0.8, and 0.5 are statistically indistinguishable from each other (for example, σ=1.2 versus 0.8: −0.003, interval (−0.007, +0.002)) and from clipping-only training, whereas σ=2.0 (ε= 22.1) is lower than σ=0.5 (σ=2.0 minus σ=0.5: −0.022, interval (−0.036, −0.007)), the first measurable loss. The apparent non-monotonic behavior of AUROC across σ in the first submission is explained by training randomness of an under-optimized model (seed-to-seed SD of 0.036 at η=0.005).

Utility is largely retained down to ε of about 22–47 but not at meaningful budgets. When σ is calibrated so that the worst client reaches ε=8, 4, 2, and 1, the AUROC is 0.553 ± 0.023, 0.524 ± 0.025, 0.515 ± 0.015, and 0.523 ± 0.012 (Table 15 and Figure 10); at ε=8, the difference between clipping-only and private training (clipping-only minus private) is +0.098, interval (+0.070, +0.127), and relative to validation-tuned non-private FedProx the private model is lower by 0.075 AUROC (private minus tuned non-private: −0.075, interval (−0.104, −0.047)), which is the cost of a meaningful budget. The operating point is also unstable at these budgets; across the five seeds, the sensitivity varies from 0.12 to 0.73 at ε=8 and from 0.08 to 0.92 at ε=4, against spans of at most 0.15 for σ between 0.5 and 1.2. Hence, the frontier is essentially flat for ε≥47, shows a small loss at ε≈22, and degrades steeply once the budget becomes small enough to be meaningful. At ε=8, HIV-PrivFed is no longer distinguishable from local-only learning (difference −0.012, interval (−0.031, +0.006)). A privacy budget of ε≥22 bounds the advantage of a membership inference adversary only trivially (Section 5), so the apparent absence of a utility cost at those budgets should not be read as a free privacy gain.

*Empirical audit.* The loss threshold membership inference audit gives an attack AUROC of 0.559 for non-private FedAvg and 0.485 for HIV-PrivFed at σ=0.8 (Table 15), that is, close to 0.5 in all cases. This attack is weak and the models are small, so this result does not demonstrate privacy, showing only that this particular attack gains little.

*Sensitivity to hyperparameters.* Table 21 varies one hyperparameter at a time around the tuned configuration. The clipping norm (C=0.5 and 2.0, AUROC 0.641 ± 0.023 and 0.650 ± 0.020), proximal weight (μ=0 and 0.1: 0.648 ± 0.020 and 0.648 ± 0.020), and number of local epochs (E=1 and 5: 0.642 ± 0.021 and 0.632 ± 0.009) change the AUROC only within about the seed-to-seed spread of the reference (0.648 ± 0.021), whereas the privacy budget moves with the number of steps (from 44.7 at E=1 to 134.7 at E=5, and over rounds from 44.7 at round 10 to 134.7 at round 50), while the AUROC at those rounds is 0.623 and 0.650. Therefore, the proximal term is not what drives the private results, which is consistent with FedProx and FedAvg being indistinguishable in the non-private setting.

*Model class and number of clients.* The model-class control behaves similarly. Differentially private logistic regression at the tuned learning rate $\eta^{*}=0.5$ reaches AUROC 0.631 ± 0.024 and 0.663 ± 0.009 at σ=2.0 and 0.8, compared with 0.672 ± 0.003 for clipping-only logistic regression and 0.647 ± 0.015 for non-private federated logistic regression (Table 18). Table 22 and Figure 11 regroup the same 1427 real participants into two, three, or five clients using prevalence-sorted groups of natural cities, random groups of cities, or IID clients. Non-private FedProx obtains AUROC 0.601 ± 0.010 with the 13 natural clients, 0.577 ± 0.012 with two prevalence-sorted clients, and 0.599 ± 0.003 with two IID clients; the private runs obtain 0.648 ± 0.021 with 13 natural clients and 0.611 ± 0.012 with two, but at a worst-client budget that falls from 92.9 to 25.5. Fewer and larger clients reduce ε markedly, as predicted by the cohort-size analysis, and the effect of natural heterogeneity on utility can be read from the difference between the natural and the IID groupings at the same *K*.

### 4.6. Client-Level Non-IID Performance and Fairness Audit

Table 23 reports performance with 95% bootstrap intervals across the 13 natural city clients for non-private FedAvg. Sensitivity varies from 0.07 in Sofia to 0.54 in Brighton, while AUROC ranges from 0.47 in Sofia to 0.78 in Verona. The intervals are wide; the median width is 0.28 for sensitivity and 0.21 for AUROC, and 4 out of the 13 cities have fewer than ten HIV-positive test participants and are flagged as unstable. The equal opportunity (sensitivity) gap is 0.47 across all cities and 0.36 across the nine stable ones, so a single global performance value conceals substantial variation between clients; however, a large part of the apparent variation between small clients is sampling noise.

Table 24 provides the same audit for HIV-PrivFed at σ=0.8 with the tuned learning rate. Its sensitivity gap is 0.48 across all cities and 0.47 across the stable cities, and its AUROC gap is 0.20 and 0.20. We report σ=0.8 because it is the middle of the sweep and its budget (ε= 92.9) lies between the extremes; all other configurations are in the supplementary results. The first submission reported that privacy narrowed the false positive rate gap while widening the AUROC gap; with the corrected training and the added intervals, the differences between the private and non-private audits are within the width of the intervals, and are not interpreted as evidence that privacy redistributes the error in either direction.

Figure 12 visualizes the intervals. The smallest client estimates should be interpreted cautiously; for example, Vilnius contains only four HIV-positive participants in the unseen test partition, so its client-level sensitivity is reported for transparency but should not be interpreted as a stable population-level estimate.

*City-specific thresholds.* Because prevalence differs between cities, a single global threshold may not be the most appropriate operating point everywhere. Table 25 and Table 26 compare the global Youden threshold with city-specific thresholds chosen on each city’s own training participants. For FedAvg, the sensitivity gap is 0.47 with the global threshold and 0.50 with city-specific thresholds, and the false positive rate gap is 0.22 and 0.33. City-specific thresholds are estimated from only 4–34 positives per city, and can be unstable; thus, they are evaluated here as an exploration as opposed to being adopted. A future deployment with larger local cohorts could calibrate them locally.

### 4.7. Statistical Robustness

Table 27 reports 95% confidence intervals obtained from 1000 stratified bootstrap resamples of the test participants, using decision thresholds derived from training data only. EasyEnsemble achieves a mean AUROC of 0.696 with a 95% confidence interval of (0.663, 0.726) and an AUPRC of 0.235 with an interval of (0.202, 0.280). Logistic regression obtains an AUROC of 0.692 with a 95% confidence interval of (0.658, 0.726) and an AUPRC of 0.213 with an interval of (0.186, 0.259).

Table 28 further compares EasyEnsemble and logistic regression using paired bootstrap resampling. The AUPRC mean difference is +0.020 with a 95% confidence interval of (−0.012, +0.055), the AUROC mean difference is +0.004 with an interval of (−0.024, +0.032), and the MCC mean difference is −0.013 with an interval of (−0.049, +0.023). Because all three intervals include zero, the observed point estimate differences on this test partition do not support a statistically significant superiority claim at the 0.05 level. Sensitivity and specificity do differ significantly (−0.084 and +0.059), but this reflects the two models operating at different points of their curves and not a difference in discrimination. Figure 13 visualizes these intervals. The bootstrap quantifies only the uncertainty due to the finite test sample; it does not include the partition-to-partition variability, which as Table 12 shows is important for this comparison.

### 4.8. Computational Efficiency and Secure Update Overhead

Table 29 summarizes the theoretical time and space complexity of the evaluated model families. The conventional linear, ensemble, neural, and federated approaches exhibit different computational scaling characteristics, while HIV-PrivFed additionally introduces differential-privacy processing, masking, and encryption operations.

Table 30 complements the theoretical analysis by showing the measured training time and response latency of the conventional classifiers and the two neural models. Logistic regression needs 0.07 ms per participant, whereas EasyEnsemble needs 82.2 ms, about 1211× more; therefore, lightweight models provide substantially lower response latency, while ensemble-based approaches require additional computation during prediction.

Table 31 summarizes the communication volume of federated training. FedAvg, FedProx, and HIV-PrivFed use exactly the same 9857-parameter model, 30 rounds, and the same 13 clients in every round, so their communication is identical. Each update occupies 39428 bytes (38.50 KiB). With 13 participating clients, each round transfers 500.55 KiB from the clients to the server and the same volume back, which amounts to 29.329 MiB in both directions over 30 rounds (14.665 MiB in the uplink alone). Differential privacy adds no communication, and the optional protections add the computation time reported next.

Table 32 reports the measured overhead of the optional protections in a single-process simulation. Each plaintext model update occupies 39,428 bytes, while the encrypted representation occupies 39,460 bytes after inclusion of the authentication tag and nonce. Pairwise masking across the 13 clients requires an average of 28.56 ms, secure aggregation requires 0.21 ms on average, and AES-GCM encryption and decryption require mean per-client latency of 0.241 ms and 0.157 ms, respectively. The total overhead, defined as masking plus aggregation plus 13 encryptions plus 13 decryptions, is 33.9 ms per communication round, and the aggregated result reproduces the unmasked mean to within a maximum absolute error of 3.6 × 10^−7^.

### 4.9. Model Summary and Testing Prioritization Utility

Figure 14 summarizes the evaluated models in two dimensions: Panel (a) places each model by AUROC and AUPRC, while panel (b) shows the operating point reached at the Youden threshold. This allows models with similar discrimination to be distinguished by the tradeoff between sensitivity and false positive rate.

To translate discrimination into the setting for which the models are intended, Table 33 shows what happens if only the highest-risk participants are tested. With a test prevalence of 10.6%, random ordering finds 30% of the positives by testing 30% of the participants, and needs 9.4 tests per positive found. Testing the top 30% of participants ranked by the centralized EasyEnsemble finds 54% of all positives (positive predictive value 19.1%, 5.2 tests per positive found), logistic regression finds 57%, non-private FedProx finds 44%, and HIV-PrivFed at σ=0.8 (see in Figure 15) finds 48%; testing the top 50% finds 73%, 76%, 63%, and 70%, respectively. The gains over random ordering are real but modest, as implied by the AUROC values below 0.70 (Section 5.4).

## 5. Discussion

This section interprets the experimental findings of HIV-PrivFed from five connected perspectives. First, it asks what the achieved Rényi-DP privacy budgets mean in practice and how they depend on the cohort size and on δ. Next, it explains where the utility loss under differential privacy comes from. It then considers client-level variation and the fairness audit, before describing how the models could and could not be used in HIV testing prioritization and which security assumptions apply. The section closes with the generalizability of the Sialon-II-based results, the consolidated limitations, and future work.

### 5.1. What the Privacy Budget Means

The achieved worst-client budgets of ε∈{22.1, 47.2, 92.9, 224.1} at $\delta =10^{-5}$ (Table 5) are far looser than the nominal ε≈1–8 targets that the noise multipliers were meant to reach, and do not provide a practically meaningful record-level guarantee. Differential privacy bounds the multiplicative change of any output probability by $e^{\varepsilon }$ and the advantage of a membership inference adversary by at most $\left(e^{\varepsilon }-1+2\delta \right)/\left(e^{\varepsilon }+1\right)$ [22,61]. This bound is 0.46, 0.76, 0.96, and 0.9993 for ε=1, 2, 4, and 8, respectively, and equals 1.00 to three decimals for every budget of 11.5 or more. At the budgets reached here, the formal guarantee excludes essentially nothing. The empirical audit points the same way as a weak sanity check, but cannot replace the guarantee. A loss threshold attack gained almost nothing even against the non-private models, for which the attack AUROC was 0.559.

The budget is high for identifiable reasons, which the present analysis separates. The budget is set by the client with the largest sampling rate (Bucharest, $n_{k}=59$, $q_{k}=0.5$), not by the largest client. It falls steeply with cohort size (92.9 at $n_{k}=59$, 20.4 at 1000, 5.2 at 10,000; Table 19), so the small clients of this federation are a first-order cause, and grows with the number of local steps (Table 21). By contrast, the choice of $\delta =10^{-5}$ matters little; it is about 70 times smaller than 1/N for the N=1,427 real training participants, as is conventionally required, and moving δ from $10^{-6}$ to $10^{-4}$ or to 1/N lowers ε by only 12% and 18%, respectively. This resolves the question of how much of the inflation is due to cohort size as opposed to batch size, epochs, or accountant configuration: all of these contribute, cohort size most strongly, and none can be removed by tuning the noise multiplier alone. Reaching ε=8 for the worst client requires σ≈4.4; at that budget, the AUROC falls to 0.553 ± 0.023 (Section 4.5). The comparison between the 13 natural clients and regrouped clients (Table 22) shows that fewer and larger clients lower ε considerably, which is why larger multi-institution cohorts are the most promising route to a meaningful guarantee (Section 5.9).

### 5.2. Source of the Utility Loss

The first submission reported that differentially private training collapsed to near-chance performance and that this collapse was a structural property of small-cohort federated learning. The corrected evaluation shows that this conclusion was largely an artifact of an untuned learning rate. With SGD at a learning rate of 0.005, clipped updates barely move the model away from its initialization, and all noise multipliers gave the same near-chance AUROC as clipping-only training (0.534 ± 0.036). Because the ratio between the accumulated signal and accumulated noise does not depend on the learning rate, a better learning rate recovers the utility without changing ε: with $\eta^{*}=0.2$, clipping-only training reaches 0.651 ± 0.010 and HIV-PrivFed reaches 0.629 ± 0.015 to 0.650 ± 0.014 for worst-client budgets of 22.1 to 224.1, clearly better than local-only learning (0.565 ± 0.005) and modestly higher than validation-tuned non-private FedProx (0.628 ± 0.007; difference +0.022). The residual is attributable to clipping, not privacy. At the tuned learning rate, clipping did not reduce utility. The noise cost was negligible for σ≤1.2, about 0.02 AUROC at σ=2.0, and large for ε≤8. Therefore, the utility cost of record-level differential privacy in this setting is small at budgets that offer no meaningful protection and large at budgets that do. The run-to-run instability reported earlier for one retraining at the middle noise level, in which the model predicted the positive class for most patients, is consistent with the same underoptimized regime. With the tuned learning rate, the operating points are stable for σ between 0.5 and 1.2 (the sensitivity varied by at most 0.15 across the five seeds), less stable at σ=2.0 (0.27), and highly unstable at the large noise multipliers needed for ε≤8 (sensitivity spans of 0.59 to 0.84 across seeds), where the threshold chosen on noisy training predictions is unreliable even though the AUROC changes little between seeds.

Two cautions apply. First, the learning-rate selection used training-derived validation data, which the (ε,δ) accounting does not cover. Second, for logistic regression, the selected value lies at the edge of the grid. The non-private arms were tuned only for the learning rate and on coarse grids; because the selected rate of the centralized ResMLP lies at the edge of its grid, the comparison of private with non-private models should be read as a statement about practical equivalence at these budgets rather than as proof that privacy or clipping improves accuracy in general.

### 5.3. Client-Level Variation, Fairness, and Thresholds

“Fairness-aware” in this paper refers to fairness-aware evaluation. No fairness term enters any training objective, and the audit consisted of per-client sensitivity, specificity, and AUROC with explicit gaps. The audit shows that while the performance of a global model varies between cities, much of the apparent variation between the smaller cities is sampling noise: with 4–35 positive test participants per city, the 95% intervals are wide, and cities with fewer than ten positives are flagged as unstable. The difference between the private and non-private audits is within the width of these intervals, so we cannot claim that privacy narrows one gap while widening another. Earlier work shows that differential privacy can have a disparate impact on groups with less data [33]. The present audit cannot exclude such an effect for small subgroups, and can only determine that it is not detectable at the client level with these sample sizes.

A single global threshold chosen by Youden’s statistic ignores the large differences in prevalence between cities. City-specific thresholds could in principle give more appropriate operating points, but have to be estimated from very few positives per city, and in our exploration change the sensitivity and false positive rate gaps only modestly and unevenly (Table 25 and Table 26). Larger local cohorts, locally calibrated thresholds, or recalibration of a shared score, are a natural step before deployment. In HIV testing prioritization, false negative and false positive errors have different but clinically meaningful consequences. False negatives may delay HIV testing, diagnosis, linkage to care, prevention counseling, and opportunities to reduce onward transmission, while false positives, although less clinically dangerous than missed cases, may increase unnecessary testing burden, anxiety, stigma, and mistrust, particularly in populations already affected by HIV-related stigma. Therefore, no single performance metric should be used to determine whether a model is acceptable for public health or clinical use; sensitivity, specificity, calibration, AUPRC, and subgroup or client-level performance should be interpreted jointly alongside privacy loss and implementation feasibility.

### 5.4. Intended Use and Practical Implications

The models in this study are not diagnostic tests and should not be used as such. Even the strongest reach an AUROC below 0.70, and at the test prevalence of 10.6% the positive predictive value is only about 19.1% when the top 30% of participants are tested (Table 33). The realistic role of the current version is as a supporting tool for testing prioritization; in a community or venue-based testing setting where tests are limited, ranking attendees by predicted risk and offering a test to those at the top first will find more infections per test than random ordering (54% of positives among the top 30% for the centralized EasyEnsemble, 44% for non-private FedProx, and 48% for HIV-PrivFed at σ=0.8 against 30% for random ordering). It could also help to decide who to approach first or to remind, though the laboratory test remains the only way to establish status. Using the score in this way would require external validation in the intended population, local calibration of the threshold and of the expected prevalence, monitoring of performance across sites, and a policy for how the information is shown to counselors and participants. The findings have several practical implications for HIV testing prioritization and privacy-preserving public health decision support [2,10].

First, the results show that collaborative learning across different sites can recover useful predictive signal compared with isolated local training. This is important for real-world HIV prevention and testing programs, since individual clinics, cities, or community-based organizations may have limited sample sizes but may collectively contain information that improves risk stratification. A federated approach allows such sites to contribute to a shared model while keeping sensitive participant-level behavioral and HIV-related data local.

Second, the results demonstrate why HIV risk prediction models should not be evaluated using accuracy alone. In HIV testing prioritization, identifying individuals who may benefit from testing, prevention counseling, or linkage to care is more important than simply maximizing overall classification accuracy. Models with high overall accuracy can still miss many HIV-positive cases, whereas models with higher sensitivity increase the false positive burden. Therefore, practical model selection should consider sensitivity, specificity, AUPRC, calibration, and client-level performance together.

Third, the study highlights the importance of fairness-aware evaluation in geographically distributed HIV screening contexts. The variation in performance across cities indicates that a model that appears acceptable overall may perform differently across local populations. For public health practice, this means that AI-based testing prioritization tools should be monitored not only at the aggregate level but across sites and subgroups. Such monitoring can help to identify where additional local calibration, threshold adjustment, or targeted outreach strategies may be needed.

Finally, the privacy–utility findings provide a practical roadmap for organizations considering privacy-preserving AI in HIV-related settings. They show that while federated training with record-level differential privacy is technically feasible and inexpensive in this setting, a meaningful guarantee requires either much larger cohorts or a willingness to accept a substantial loss of accuracy. Health systems and community programs should treat privacy, accuracy, sensitivity, fairness, and computational cost as joint design criteria rather than separate technical concerns, and when describing a system as private, should state the achieved privacy budget as opposed to the noise multiplier.

### 5.5. Statistical Uncertainty and Claims We Do Not Make

A paired bootstrap comparison (Table 28) between our two strongest conventional models—an imbalance-aware ensemble and a simple logistic regression—shows a mean AUPRC difference of +0.020 with a 95% confidence interval of (−0.012, +0.055), which includes zero. Therefore, we do not claim that the ensemble is definitively superior on the original partition. Repeated resampling of the partition (Table 12) nevertheless shows a modest and consistent advantage of the ensemble in terms of AUROC; logistic regression remains a simpler and computationally more efficient alternative, with discrimination within about 0.03 AUROC. More broadly, every performance figure in this paper should not be read in isolation but alongside its confidence interval or standard deviation. We do not claim that federated learning matches centralized learning with the same model class, that differential privacy at the budgets reached here protects participants, or that the results transfer to other populations.

### 5.6. Security Assumptions

The privacy and security mechanisms address different threats, which we have kept separate throughout (Section 3.4.8 and Table 6). Differential privacy addresses an honest-but-curious server and an analyst who holds the final model, with the strength of the guarantee set by the achieved ε, and hence weak here. Secure aggregation and AES-GCM were benchmarked only as a simulation, and address a server that inspects individual updates and a passive eavesdropper, respectively. Malicious clients, poisoning and backdoor attacks, collusion, active attacks on the protocol, gradient inversion without effective noise, client dropout, key management, and side channels are not covered, and the framework should not be described as secure against them.

### 5.7. Generalizability

Sialon-II is a real peer-reviewed behavioral risk cohort of men who have sex with men across 13 European cities; its demographic composition, risk factor distribution, and HIV prevalence (4.3%–30.5% across cities) are specific to that population and region. We make no claim that HIV-PrivFed’s absolute performance figures transfer to other populations, risk groups, or geographies without re-validation, though the methodology consisting of natural non-IID client construction, honest privacy accounting, and the full utility/fairness/efficiency measurement suite is population-agnostic and directly reusable. Five features of the cohort limit transfer in particular. First, the participants were recruited through venue-based time–location sampling and respondent-driven sampling, which are not probability samples of all MSM, so selection effects may shape both prevalence and the associations learned. Second, the outcome prevalence (10.6% in the test partition, 2.5%–19.8% across cities) determines the positive predictive value of any risk score; a population with a lower prevalence would see more false alarms per true case at the same sensitivity and specificity. Third, the data were collected in 2013–2014; HIV prevention and testing practice, including the availability of pre-exposure prophylaxis, has changed since then, so the behavioral associations may have shifted. Fourth, the 51 features are drawn from a behavioral questionnaire designed for this population; other groups (for example heterosexual cohorts, people who inject drugs, or transgender populations) and other settings (for example antenatal or general population screening) involve different risk factors and different data sources, so the feature set itself would have to change. Fifth, the 13 clients are cities in one study with a common protocol, whereas real clinical sites differ in questionnaires, coding, and workflows. Extension to other populations and clinical settings would require independent external validation on cohorts representative of those groups, and a model trained on this cohort should not be applied to them without it.

### 5.8. Limitations

The limitations of the present work are consolidated below:

1. Single cohort and period. All results come from one MSM cohort collected in 2013–2014 (Section 5.7); there is no external or prospective validation and no analysis of clinical or cost-effectiveness outcomes.
2. One primary partition. The primary results use one 50:50 participant-level partition. Split dependence was quantified only for two conventional models on the 3785 real participants available in the processed files (Table 12); the federated and private models were not repeated over partitions.
3. Undersampled clients. The federated clients hold 59–127 participants, with 931 negative participants removed by undersampling and not included in the processed files. Experiments on the full 2358-participant real training partition ($n_{k}\approx 181$) were not possible, and the effect of the undersampling on utility remains confounded with the small-cohort setting. For the privacy budget, the effect can be computed without training; with the full-partition client sizes (taken from the test sizes), the worst-client budget at σ=0.8 would be 76.6 instead of 92.9, so the undersampling explains only a minor part of the inflated budgets (Table 19).
4. Small client samples. Client-level estimates rest on 4–35 positive test participants per city. Cities with fewer than ten are flagged as unstable, and even the stable ones have wide intervals (Table 23 and Table 24).
5. Weak formal privacy. The achieved budgets ε≥ 22.1 give no practically meaningful record-level guarantee (Section 4.5). Only one accountant family (Rényi DP, with PRV for comparison) was used. The guarantee is nominal and restricted to the clipped and noised gradient releases; the class weights, aggregation weights, pooled threshold, pooled z-score statistics, and learning rate selection on training-derived validation data are outside the accounting (Section 3.4.4). The membership inference audit is a weak loss threshold attack. The unit of privacy is one record per participant, not a site. Pooled feature means and variances are used for z-scoring, which a real federation would have to compute securely.
6. Simulated system layer. Secure aggregation and AES-GCM were simulated in one process to measure overhead; they contain no key agreement, no dropout handling, and no authentication, and should not be taken as a security guarantee (Section 3.4.8).
7. Limited tuning. The conventional classifiers were not tuned. For the neural and federated models, both private and non-private, only the learning rate was tuned on inner validation data using coarse grids; for the logistic regression and centralized ResMLP, the selected value lies at the edge of the grid, so an even larger value might do better. Local epochs, rounds, and batch size were varied only one at a time.
8. Fairness is audited, not enforced. Fairness was assessed after training and by city only, and no fairness term entered any objective. Subgroups within cities, calibration by city, and intersectional groups were not examined.
9. Approximate synthetic data. The city-conditioned SMOTENC samples are distinguishable from the real ones and shift the feature–label associations (Section Fidelity of the Synthetic Positives); conclusions about augmentation apply to this generator only.
10. Statistical scope. Bootstrap intervals quantify test sample uncertainty, while the standard deviations quantify training randomness over five or three seeds; neither captures all sources of variability, and no correction for multiple comparisons was applied.
11. Modest discrimination. Even the best models reach AUROC below 0.70, so the models are not suitable as standalone diagnostic tools (Section 5.4).

### 5.9. Future Work

The baselines that the earlier version of this study identified as most informative, namely, federated and differentially private federated logistic regression and a clipping-only ablation, are now part of the evaluation. The remaining priorities are outlined below. (i) Repeat the analysis on the full 2358-participant real training partition and on larger federations, as the cohort-size analysis shows that the achieved ε falls steeply with client size; assemble larger federated cohorts through multi-institution or multi-country data-sharing consortia. (ii) Reach meaningful budgets (for example, ε≤10) with fewer local steps per round, smaller batches, more careful subsampling, and tighter accounting, then evaluate the remaining utility. (iii) Use adaptive per-client clipping norms tuned to each city’s gradient scale rather than a single global constant. (iv) Add personalized or clustered federated learning that lets cities with similar risk profiles share statistical strength without forcing a single global model onto genuinely heterogeneous populations. (v) Move from fairness auditing to fairness-constrained federated training [31] and extend the audit to demographic and social-vulnerability subgroups nested inside cities (age, education, migration status, sexual orientation outness, and other available variables). This is necessary because city-level aggregates can conceal disparities and DP-SGD noise is not applied in proportion to effective subgroup sample size, smaller or marginalized subgroups may absorb a disproportionate share of the utility cost [33]. (vi) Replace the simulated system layer by a complete secure aggregation protocol with key agreement, dropout handling, and client authentication, then test stronger privacy attacks, including poisoning and inversion attacks. (vii) Perform external and prospective validation on other populations and settings. (viii) Explore federated multi-view clustering [45,46,47] for unsupervised discovery of risk phenotypes across sites or when behavioral, clinical, and laboratory views of the same participants are held by different institutions.

## 6. Conclusions

This study has presented HIV-PrivFed, a privacy–utility–fairness-aware federated learning framework for HIV screening under natural non-IID clinical heterogeneity. HIV-PrivFed audits fairness during evaluation, and fairness does not enter the training objective. Using the Sialon-II dataset, the 13 participating European cities were retained as real federated clients rather as opposed to being generated through artificial data partitioning. A comprehensive evaluation was conducted across ten conventional machine learning models, a centralized residual MLP, local-only learning, FedAvg, FedProx, and differentially private HIV-PrivFed configurations under one fully specified protocol. Among the conventional approaches, the real-only EasyEnsemble achieved the highest point estimates, with an AUROC of 0.6956 and an AUPRC of 0.2355, while the paired bootstrap analysis on the original partition showed no statistically significant difference from logistic regression; repeated resampling of the partition indicated a modest but consistent advantage of EasyEnsemble (mean AUROC difference +0.032). In the federated setting, FedAvg and FedProx improved predictive performance relative to isolated local training, with FedProx achieving an AUROC of 0.607 ± 0.011, compared with 0.565 ± 0.005 for local-only learning; a model-class control showed that federated logistic regression (AUROC 0.647 ± 0.015) outperformed the federated residual MLP under the same Adam protocol, although this gap largely disappeared once the ResMLP was trained with a tuned learning rate. These results confirm that collaborative learning can recover useful cross-client information even when the participating populations exhibit substantial differences in cohort size, HIV prevalence, and behavioral risk distributions.

The privacy-aware experiments led to a more nuanced conclusion than our first submission. With per-client Rényi accounting, the achieved worst-client privacy budgets were ε= 22.1 to 224.1 at $\delta =10^{-5}$, far looser than the nominal targets and without a practically meaningful record-level guarantee; the smallest client determines the guarantee, and the budget falls steeply with cohort size. Once the learning rate was tuned on training-only validation data, the private models retained most of their accuracy at these budgets (AUROC 0.629 ± 0.015 to 0.650 ± 0.014, against 0.651 ± 0.010 for clipping-only training, with a small loss only at the smallest of them), modestly above validation-tuned non-private FedProx (0.628 ± 0.007). This residual is attributable to clipping, but accuracy fell to 0.553 ± 0.023 and 0.524 ± 0.025 when the noise was calibrated to worst-client budgets of 8 and 4. Therefore, in this small-cohort setting useful accuracy and a meaningful privacy guarantee did not coexist. Client-level estimates were highly uncertain, with several cities contributing fewer than ten positive test participants, and the audit did not detect a clear effect of privacy on cross-city disparities. The optional secure aggregation and AES-GCM protections were benchmarked in a single-process simulation and added a measured overhead of 33.9 ms per communication round, but should not be read as a deployed security guarantee. Overall, the findings show that privacy-preserving HIV screening cannot be assessed through predictive accuracy alone; privacy loss, minority class detection, client heterogeneity, fairness, calibration, and computational overhead must be considered jointly. A key limitation of this study is its exclusive reliance on the Sialon-II MSM cohort. Because participants were recruited through venue- and network-based sampling of MSM communities rather than general population or clinical screening pathways, the reported privacy–utility trade-offs and predictive performance are most directly generalizable to MSM and similar community-based testing contexts; extension to other populations or settings would require independent external validation. Future work will focus on larger multi-institutional cohorts, tighter privacy accounting and meaningful budgets, adaptive client-specific clipping, personalized or clustered federated learning, fairness-constrained training, and a complete secure aggregation protocol.

## Figures and Tables

**Figure 1:**
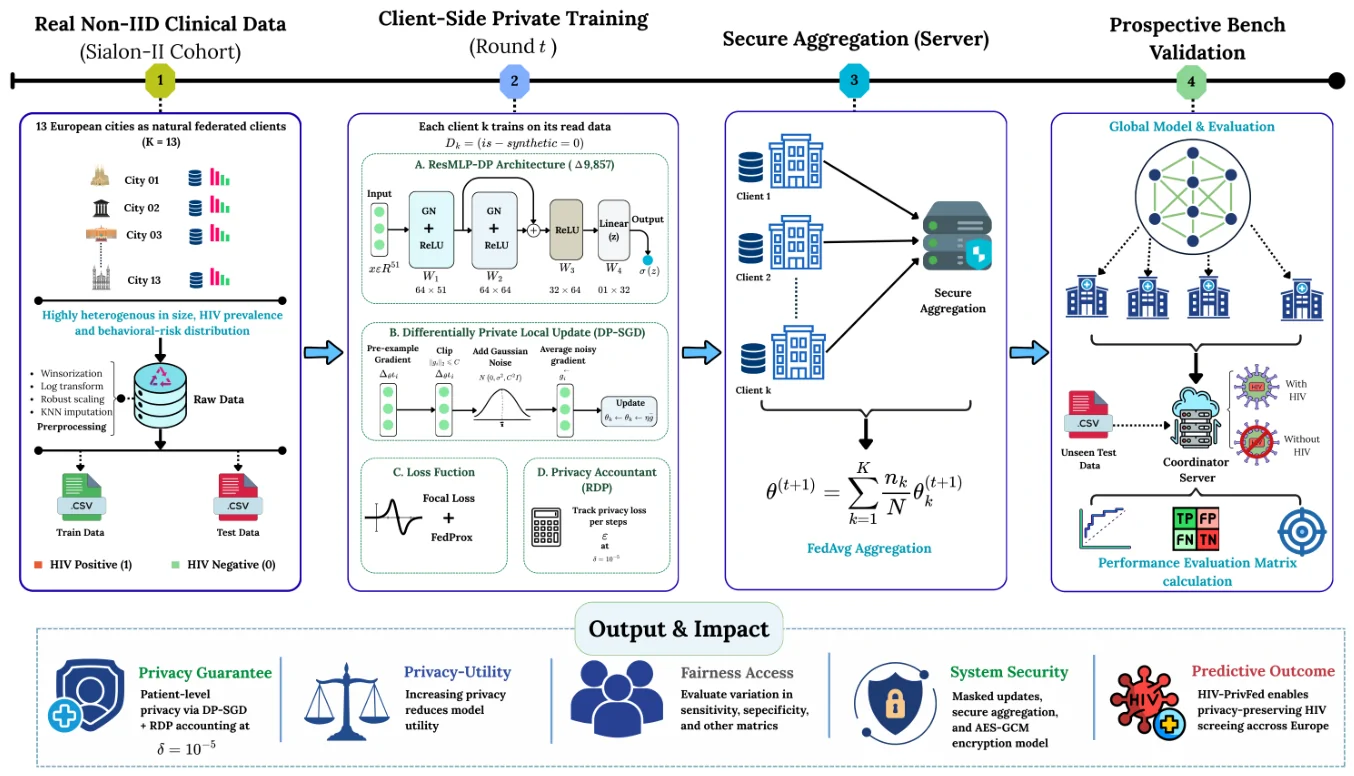
Overview of the HIV-PrivFed pipeline. (1) Real non-IID clinical data from the Sialon-II cohort span K=13 European cities as natural federated clients with differences in size, HIV prevalence, and behavioral risk profile. Records are Winsorized, log-transformed, robustly scaled, and KNN-imputed before an HIV-positive/negative train–test split. (2) In client-side private training (round *t*), each client trains locally on real data $D_{k}$ via (A) a compact ResMLP-DP network (GroupNorm+ReLU residual blocks, ≈9.8 K parameters), (B) DP-SGD, which clips per-example gradients ($∥g_{i}{∥}_{2}\leq C$), adds Gaussian noise, and averages them into the privatized gradient $\tilde{g}$ before the update $\theta \leftarrow \theta -\eta (\tilde{g}+\mu \left(\theta -\theta^{\left(t\right)}\right))$ (Equations (20)–(22)), (C) a focal loss and FedProx objective, and (D) an RDP accountant bounding privacy loss ε at $\delta =10^{-5}$. (3) A server combines client updates via FedAvg, $\theta^{\left(t+1\right)}=\sum_{k=1}^{K}\frac{n_{k}}{N}\theta_{k}^{\left(t+1\right)}$; secure aggregation and AES-GCM encryption are optional simulated system-layer protections. (4) The global model is evaluated on the disjoint patient-level held-out Sialon-II test partition, yielding a TP/FP/FN/TN confusion matrix. The bottom panel highlights system-wide properties: the privacy guarantee via DP-SGD with RDP accounting, the privacy–utility trade-off, fairness-aware evaluation across sensitivity and specificity, system security through the simulated protections, and the predictive outcome for privacy-preserving HIV screening across Europe.

**Figure 2:**
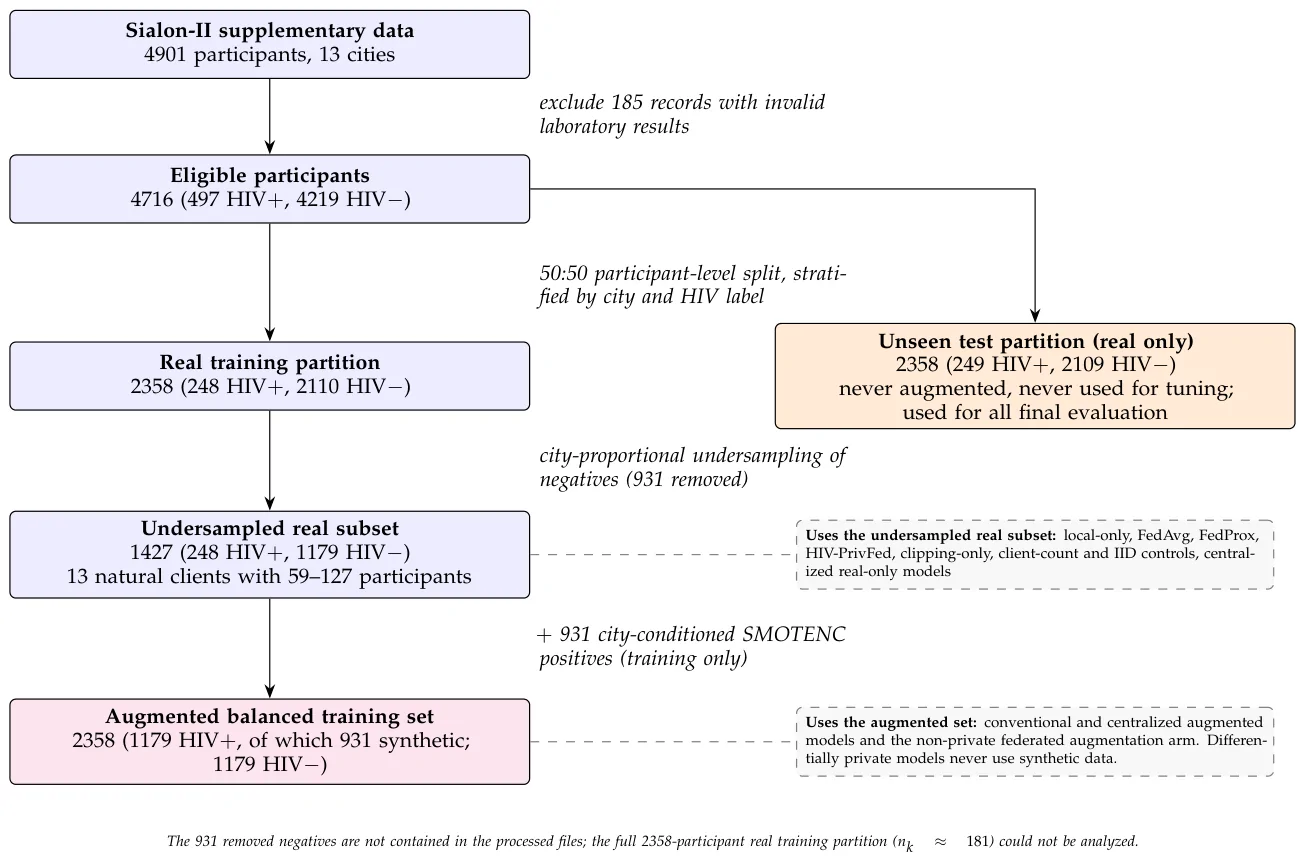
Data flow and training populations. Blue: real participants; magenta: synthetic positives (training only); orange: held-out evaluation set. Every reported model is evaluated on the same unseen test partition.

**Figure 3:**
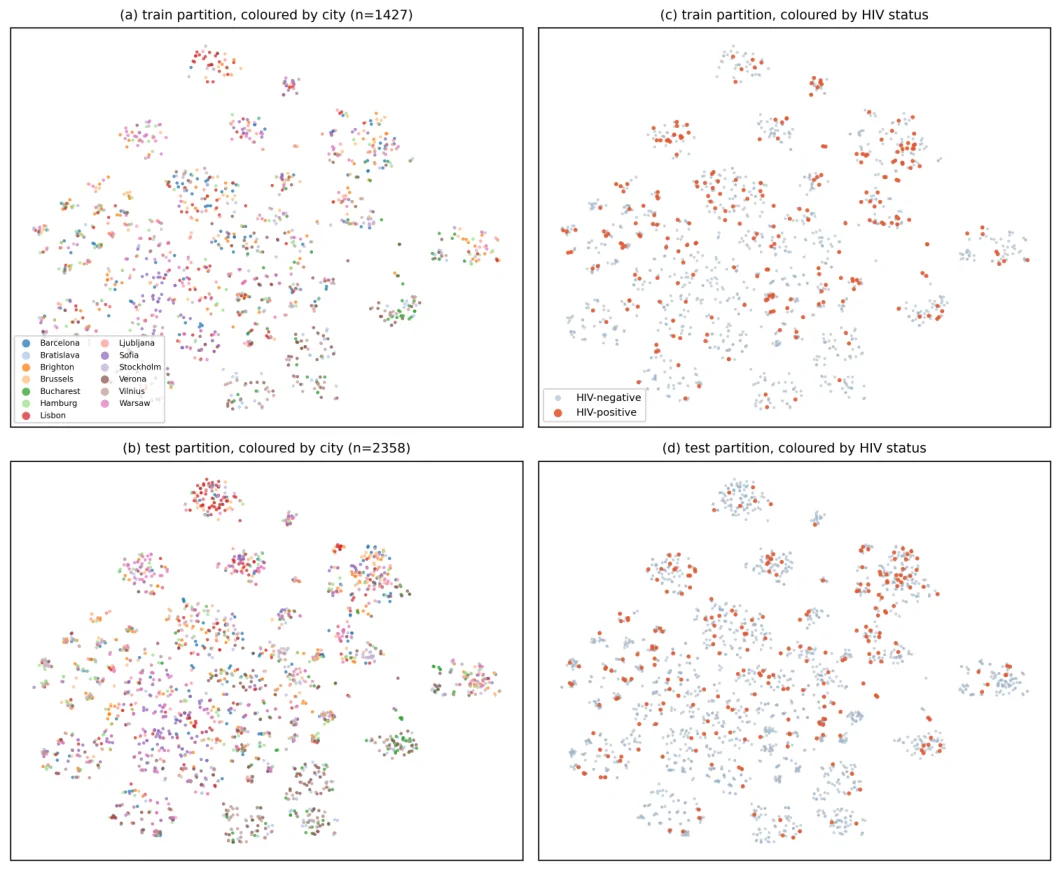
Exploratory two-dimensional t-SNE embedding of the 3785 real participants available in the processed files (1427 training participants, top row; 2358 test participants, bottom row), colored by city (**left**) and HIV status (**right**). The embedding is shown only to illustrate feature-space heterogeneity, and is not evidence of fairness or predictive performance; the 931 negative participants removed by undersampling are not in the processed files, so the embedding contains 3785 participants as opposed to 4716 and has no panel for the augmented training set. Quantitative shift measures are given in Table 10.

**Figure 4:**
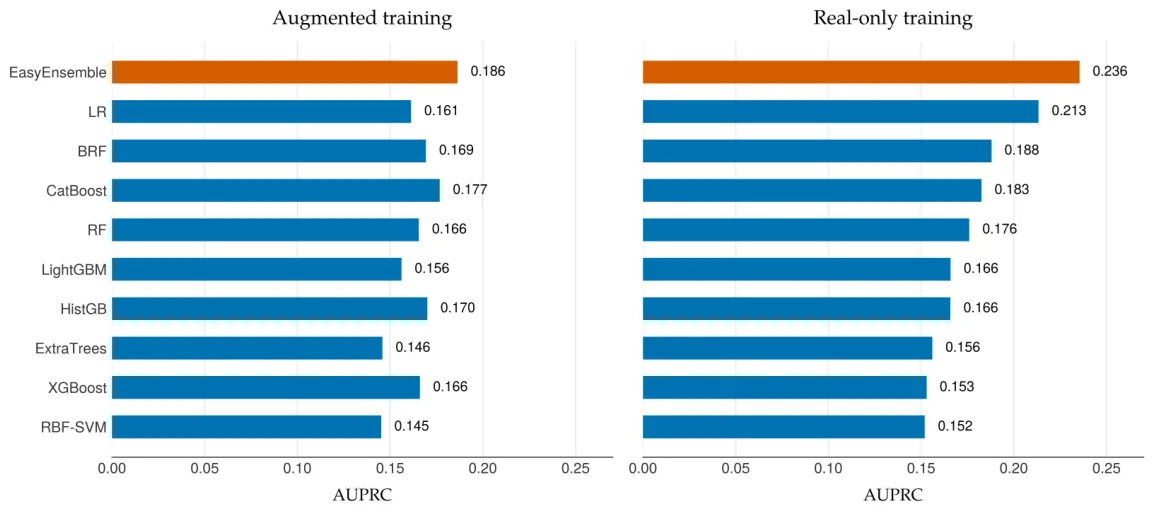
AUPRC of the ten conventional classifiers trained on augmented data (**left**) and on real-only data (**right**) and evaluated on the unseen test set (n = 2358; prevalence 10.6%). AUPRC does not depend on the decision threshold.

**Figure 5:**
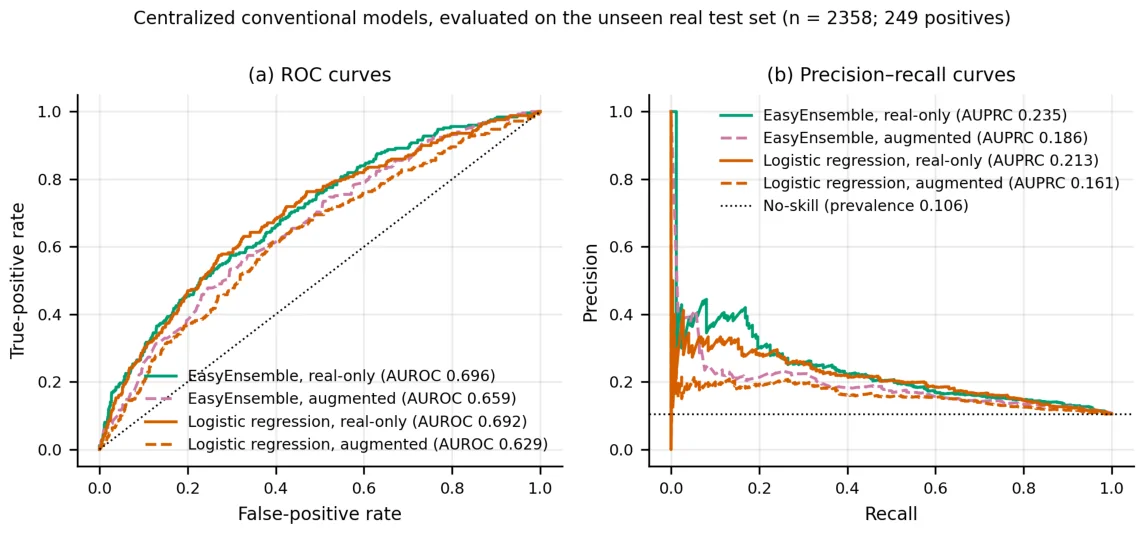
Receiver operating characteristic (**a**) and precision–recall (**b**) curves of centralized EasyEnsemble and logistic regression, each trained on real-only data (solid) or augmented data (dashed) on the unseen test set (n = 2358; 249 positives). Single fits with seed 42; dotted lines show the no-skill reference.

**Figure 6:**
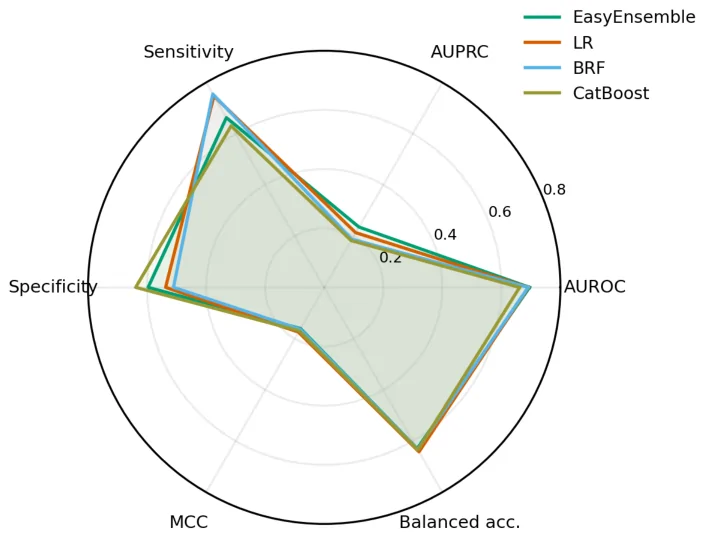
Multi-metric profile of the four strongest real-only centralized classifiers on the unseen test set (AUROC, AUPRC, sensitivity, specificity, MCC, and balanced accuracy). Sensitivity, specificity, MCC, and balanced accuracy use out-of-fold training thresholds.

**Figure 7:**
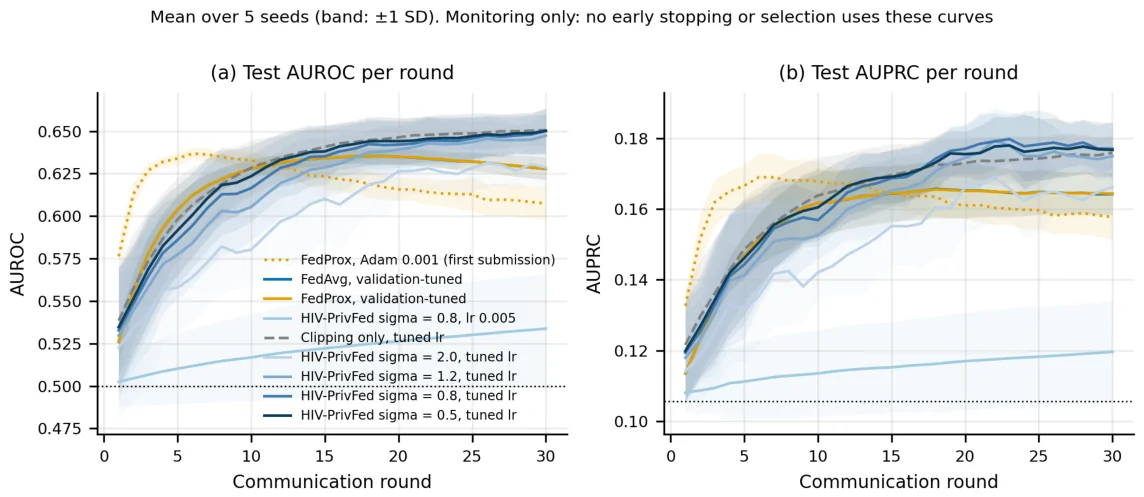
Test AUROC (**a**) and AUPRC (**b**) after each communication round for non-private FedProx with the learning rate of the first submission and for validation-tuned FedAvg and FedProx, for HIV-PrivFed at sigma = 0.8 with the learning rate of the first submission (0.005), for clipping-only training with the tuned learning rate, and for HIV-PrivFed with the tuned learning rate at four noise multipliers. Lines: mean over five seeds; bands: ±1 SD. The curves are monitored on the test set for display only; no stopping or model selection uses them.

**Figure 8:**
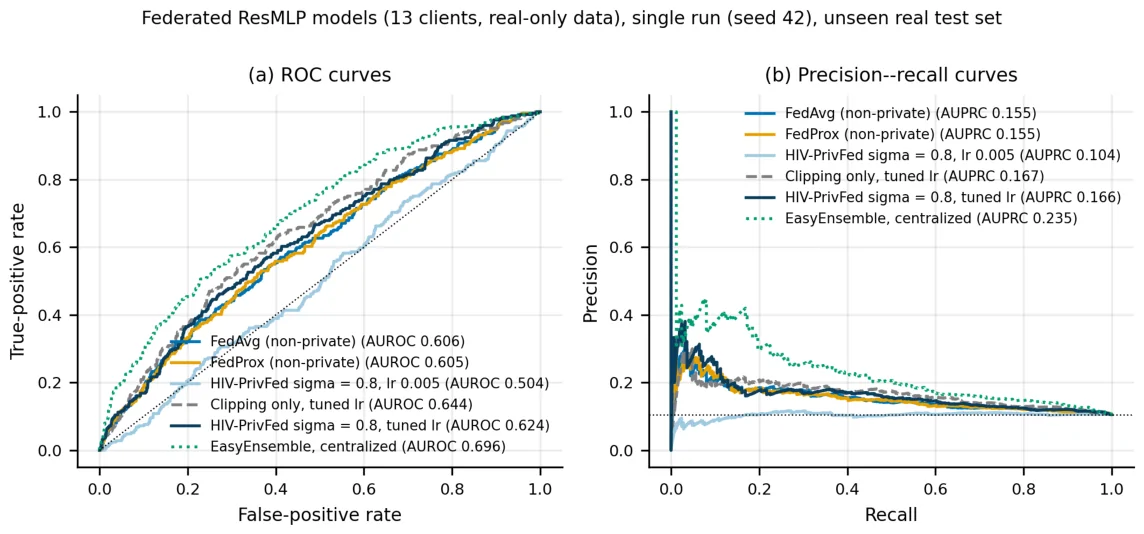
ROC (**a**) and precision–recall (**b**) curves of the federated ResMLP models trained on the undersampled real subset (13 clients) and evaluated on the unseen test set: non-private FedAvg and FedProx, HIV-PrivFed at sigma = 0.8 with learning rate 0.005, and clipping-only and HIV-PrivFed at sigma = 0.8 with the tuned learning rate. Centralized real-only EasyEnsemble is the dotted reference. Single run (seed 42).

**Figure 9:**
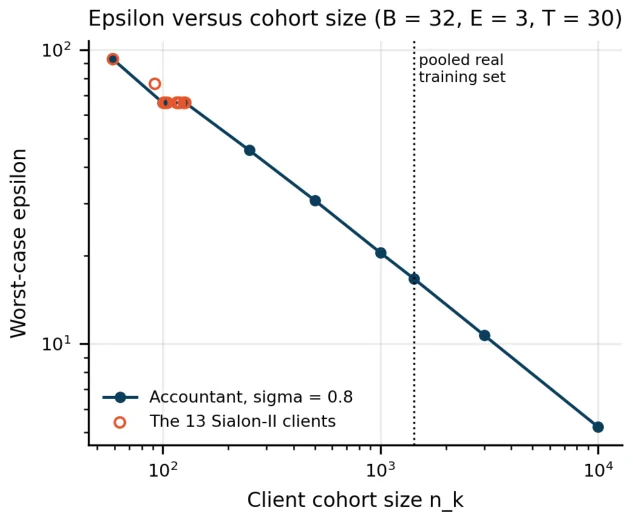
Worst-case privacy budget ε (RDP, $\delta =10^{-5}$, σ=0.8, batch parameter 32, three local epochs, 30 rounds) as a function of the cohort size of a client (line) and for the 13 Sialon-II clients (circles). The vertical dotted line marks the whole undersampled real subset treated as one client.

**Figure 10:**
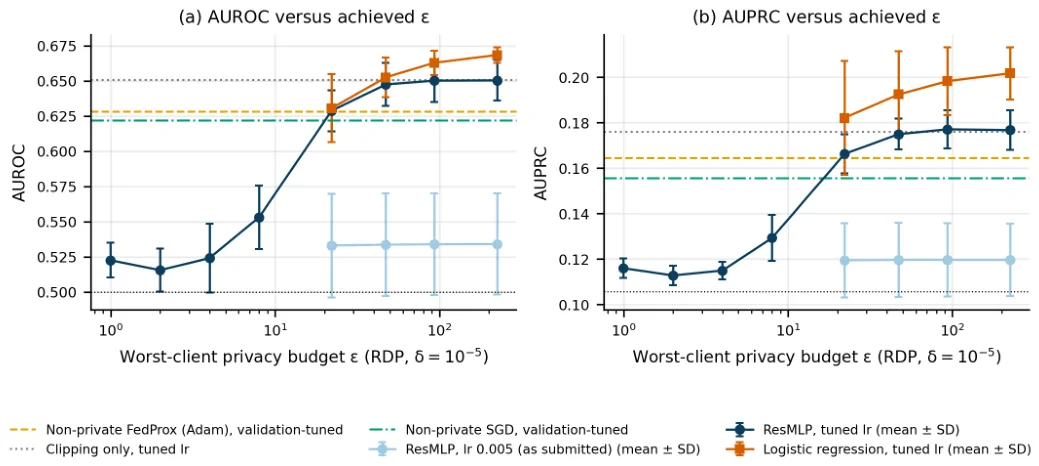
Test AUROC (**a**) and AUPRC (**b**) of HIV-PrivFed as a function of the worst-client privacy budget ε (RDP, $\delta =10^{-5}$; logarithmic axis) for ResMLP with the first-submission learning rate (0.005), for ResMLP with the tuned learning rate (including noise multipliers calibrated to ε of 1, 2, 4, and 8), and for logistic regression with its tuned learning rate. Points: mean over five seeds; bars: ±1 SD. Horizontal lines: validation-tuned non-private FedProx (Adam), clipping-only at the tuned learning rate, and validation-tuned non-private SGD; dotted: chance (AUROC) or prevalence (AUPRC).

**Figure 11:**
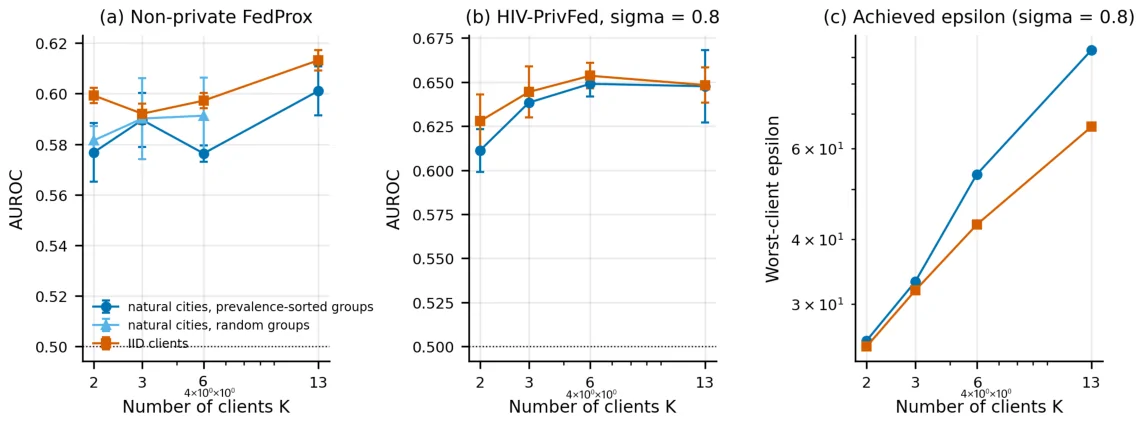
Effect of the number of clients *K* on test AUROC for non-private FedProx (**a**) and HIV-PrivFed at σ=0.8 (**b**) and on the worst-client privacy budget (**c**). The same 1427 real training participants are grouped into *K* clients as prevalence-sorted groups of natural cities, random groups of cities, or IID clients; K=13 uses the natural cities. Mean ± SD over three seeds.

**Figure 12:**
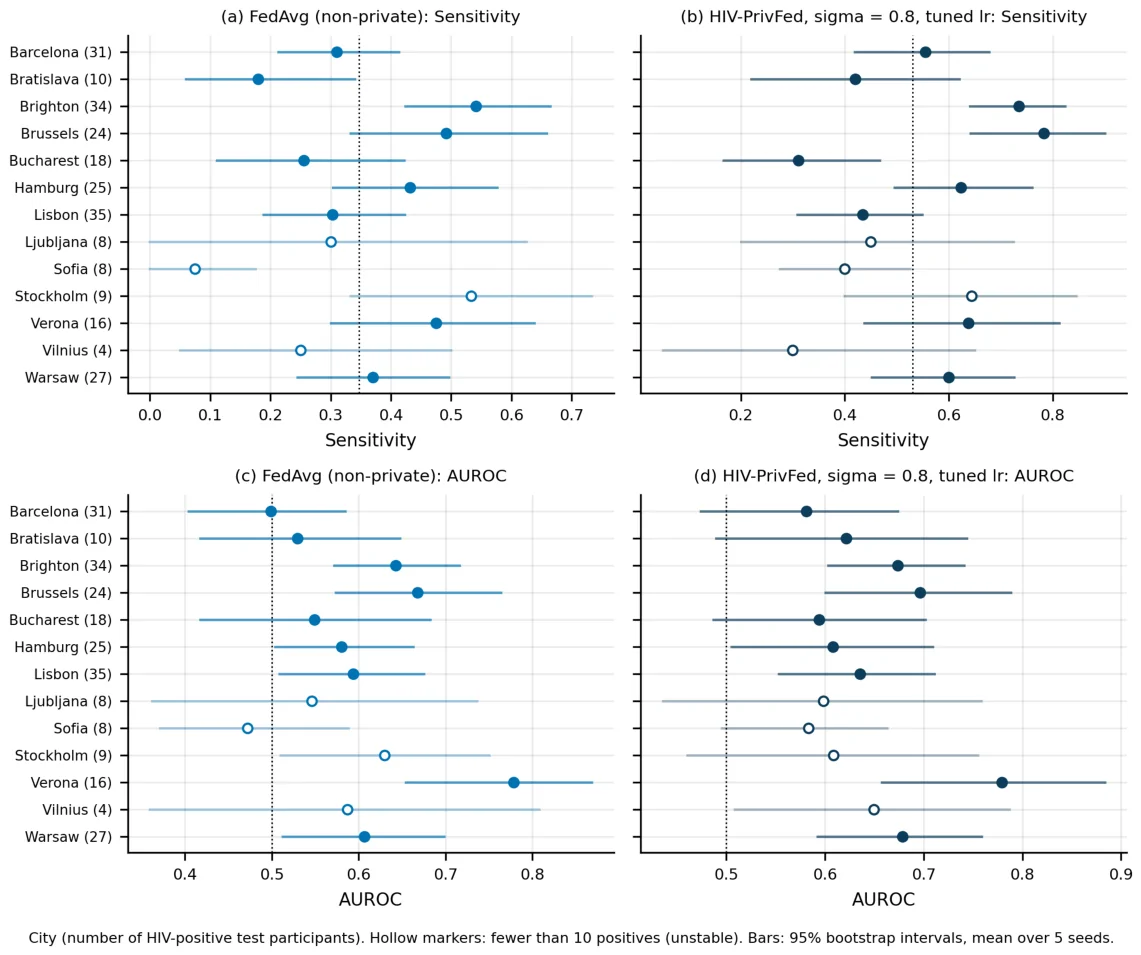
Client-level sensitivity (**top**) and AUROC (**bottom**) with 95% bootstrap intervals for non-private FedAvg (**left**) and HIV-PrivFed at σ=0.8 with the tuned learning rate (**right**). Labels give the number of HIV-positive test participants; hollow markers denote cities with fewer than ten positives (unstable). Means over five seeds; dotted lines: chance level (AUROC) or the mean over cities (sensitivity).

**Figure 13:**
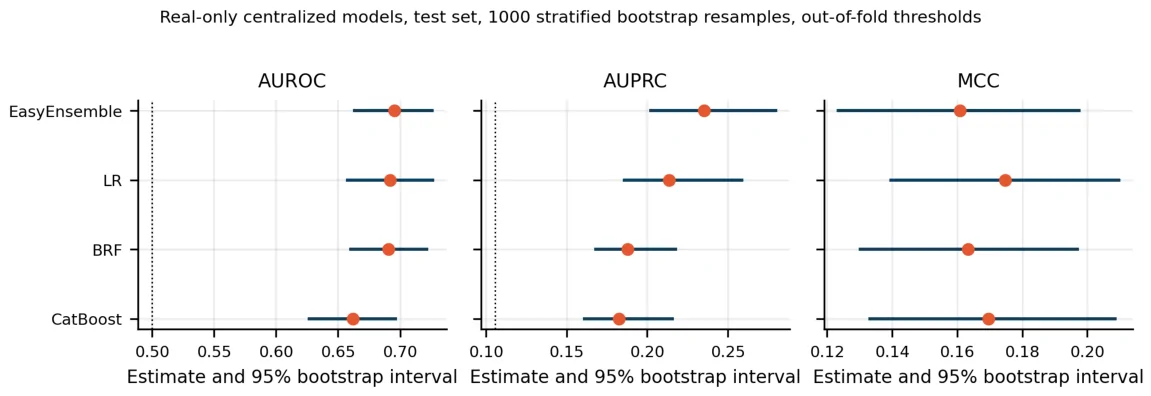
Bootstrap 95% confidence intervals (1000 stratified resamples of the test set) for AUROC, AUPRC, and MCC of the four strongest real-only centralized models, with out-of-fold training thresholds. Overlapping intervals are consistent with the paired-bootstrap result in Table 28.

**Figure 14:**
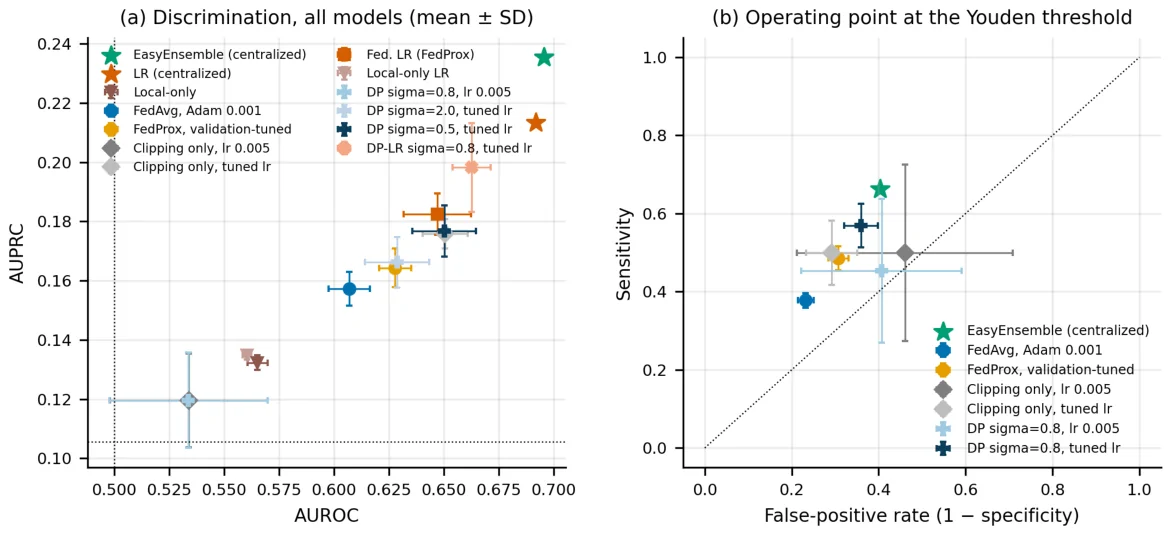
Two-dimensional summary of the evaluated models on the unseen test set: (**a**) AUROC versus AUPRC (mean ± SD over seeds for federated and neural models, with single fits for the centralized models shown as stars) and (**b**) operating points at the Youden threshold, showing false positive rate versus sensitivity for selected models. Non-private arms use the validation-tuned learning rate except where the learning rate is stated.

**Figure 15:**
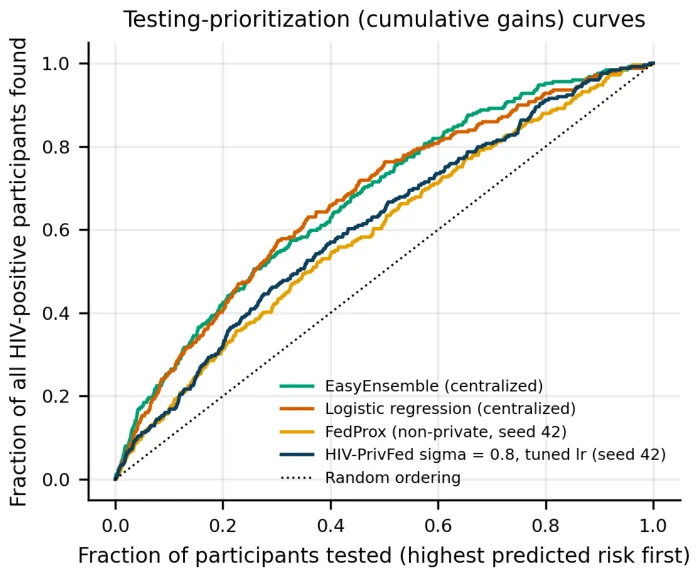
Cumulative gains: share of all HIV-positive test participants found when participants are tested in order of decreasing predicted risk, for centralized EasyEnsemble and logistic regression and for non-private FedProx and HIV-PrivFed (σ=0.8, tuned learning rate; seed 42). The dotted diagonal is random ordering.

**Table 1:** Roles of the evaluated components. Each component has exactly one role.

Component	Role	Acts on	Protection Provided	Part of HIV-PrivFed?
Ten conventional classifiers	Baseline (centralized)	Pooled data	None	No
Centralized ResMLP	Baseline (centralized)	Pooled data	None	No
Local-only learning	Baseline (no collaboration)	Each client alone	Nothing is shared	No
FedAvg	Baseline (federated)	Clients and server	Raw data stay local; updates are not protected	No
FedProx (proximal term)	Federated algorithm	Client objective	As FedAvg	Yes (non-private core)
Class-weighted focal loss	Training objective	Client objective	None	Yes
DP-SGD (clipping and Gaussian noise)	Record-level privacy	Client update	Nominal (ε,δ)-DP for the clipped and noised gradient releases only; other quantities are outside the accounting (Section 3.4.8)	Yes (private core)
Rényi-DP accountant	Privacy accounting	Per client	Quantifies ε at fixed δ	Yes
Secure aggregation (pairwise masking)	System layer (simulated)	Client–server	Server learns only the sum of updates, if fully implemented	Optional; overhead benchmarked
AES-GCM	System layer (simulated)	Channel	Confidentiality and integrity in transit; no record-level guarantee	Optional; overhead benchmarked
Per-client fairness audit	Evaluation protocol	Test predictions	None	No (not in the loss)

**Table 2:** Training population used by each experimental family. “pos.” = HIV-positive participants.

Experimental Family	Training Data	Participants	Clients	Synthetic Data	DP Guarantee Covers
Conventional classifiers, real-only	Undersampled real subset	1427 (248 pos.)	Pooled	No	n/a
Conventional classifiers, augmented	Augmented set	2358 (1179 pos.)	Pooled	Yes (931)	n/a
Centralized ResMLP, real-only/augmented	As above	1427/2358	Pooled	No/Yes	n/a
Local-only ResMLP	Undersampled real subset	59–127 per client	13	No	n/a
FedAvg, FedProx (non-private)	Undersampled real subset	1427	13	No	n/a
Federated augmentation arm (non-private)	Augmented set, per city	2358	13	Yes (per-city)	n/a
HIV-PrivFed, clipping-only, SGD controls	Undersampled real subset	1427	13	No	Real participants
Client-count and IID controls	Undersampled real subset, regrouped	1427	2, 3, 5, or 13	No	Real participants
All final evaluation	Unseen test partition	2358 (249 pos.)	–	No	–
*Not performed*	Full real training partition	2358 ($n_{k}\approx 181$)	13	No	Data not available

**Table 3:** Fidelity audit of the 931 synthetic HIV-positive samples against the 248 real HIV-positive samples.

Check	Result	Reference Value
Maximum |SMD|, four numeric columns	0.036	near 0 = marginals agree
Exact duplicates of real positives	0	0 expected
Pearson correlation of pairwise correlations (44 columns)	0.865	two real halves: 0.807
Mean absolute difference of pairwise correlations	0.079	two real halves: 0.097
Real-versus-synthetic discriminator AUROC (logistic/forest)	0.701/0.824	0.5 = indistinguishable
Median nearest-real distance: synthetic versus real positives	2.11 vs. 4.95	smaller = lower dispersion
Binary columns: maximum/mean difference of proportions	0.197/0.053	–
Feature–label association vs. real test set (Spearman): real-only/augmented	0.789/0.523	higher = closer to real test
Top-10 features by association also in test top-10: real-only/augmented	9/7	–

**Table 4:** Notation used in the equations, algorithms, and Figure 1. Lowercase *ℓ* is always the loss of one example and calligraphic J the objective of one client.

Symbol	Meaning
*K*, *k*	Number of clients (13) and client index
$D_{k}$, $n_{k}$, $n_{k}^{+}$, $n_{k}^{-}$, *N*	Real data of client *k*; its size, number of positives and negatives; $N=\sum_{k}n_{k}$
*d*; $x_{i}$, $y_{i}$	Input dimension (51); feature vector and label ($y_{i}\in \left\{0,1\right\}$) of participant *i*
θ; $\theta^{\left(t\right)}$; $\theta_{k}$	Model parameters; global parameters broadcast at round *t*; parameters of client *k*
$z_{i}$; $p_{i}=\mathrm{sig}\left(z_{i}\right)$	Logit and predicted probability; sig is the logistic function (σ is never used for it)
$p_{i}^{★}$	Probability assigned to the true class of participant *i*
$w_{k}=n_{k}^{-}/n_{k}^{+}$; γ	Positive-class weight of client *k*; focal exponent (γ=2)
$\ell_{i}\left(\theta \right)$; $\ell_{i}^{\mathrm{BCE}}$	Loss of one example (focal); its class-weighted binary cross-entropy factor
$J_{k}\left(\theta \right)$	Objective of client *k*: mean example loss plus the proximal term
*T*, *t*; *E*	Communication rounds and round index; local epochs per round
$B_{k}$; $q_{k}$; S; ${\bar{b}}_{k}$	Batch-size parameter $\min(32,n_{k}-1)$; Poisson sampling rate $1/\lceil n_{k}/B_{k}\rceil$; sampled lot; expected lot size $q_{k}n_{k}$
$g_{i}$; ${\bar{g}}_{i}$; $\tilde{g}$	Per-example gradient; clipped per-example gradient; privatized (noised, averaged) lot gradient
*C*; σ; η; μ	Clipping norm; noise multiplier; learning rate; proximal weight
ε; δ	Privacy budget and failure probability of (ε,δ)-DP ($\delta =10^{-5}$)
α; $\varepsilon_{\mathrm{RDP}}\left(\alpha \right)$; $A_{k}$	Rényi order; Rényi-DP curve; accountant of client *k*
ξ	Small numerical constant in preprocessing (unrelated to privacy)
$a_{ik}$; $N_{7}\left(i\right)$	Inverse-distance weight and seven-nearest-neighbor set used for imputation
$\tau^{*}$; TPR, FPR	Youden decision threshold; true-positive and false-positive rate

**Table 5:** Mapping from the noise multiplier to the nominal target and the achieved patient-level privacy budget.

σ	Nominal ε	Worst-Client ε (RDP)	Selected α	ε Range over Clients	Worst-Client ε (PRV)	Engine Check
2.0	≈1	22.09	2.2	14.61–22.09	20.63	22.09
1.2	≈2	47.18	1.7	31.75–47.18	44.23	47.18
0.8	≈4	92.93	1.4	66.05–92.93	87.63	92.93
0.5	≈8	224.10	1.2	178.39–224.10	212.98	224.10

Schedule: $B_{k}=\min\left(32,n_{k}-1\right)$, Poisson sampling, E=3, T=30, $\delta =10^{-5}$. The worst client is Bucharest ($n_{k}=59$, $q_{k}=0.5$, 180 steps); of 12 further clients, 11 have $q_{k}=0.25$ (360 steps) and Vilnius has $q_{k}=1/3$ (270 steps). The nominal target ε is the value a conventional single-client design rule would suggest; it is not achieved. PRV: Opacus PRV accountant, shown because it is tighter than RDP. Engine check: mean of the worst-client ε read from the accountants during the 5-seed runs.

**Table 6:** Security assumptions, covering which threats are and are not covered by each protection.

Threat	Covered?	Mechanism	Residual Risk or Limitation
Honest-but-curious server inspecting every update (A1)	Nominal only	DP-SGD bounds leakage through the gradient releases only; secure aggregation (if fully implemented) hides single updates	Not meaningful at the achieved ε (Table 5); our masking is a simulation
Passive eavesdropper on the channel (A2)	Yes	AES-GCM authenticated encryption	Key management and authentication not implemented
Membership inference on the released model (A3)	Nominal only	DP-SGD; empirical loss-threshold audit (Section 4.5)	Not meaningful at the achieved ε; weak attack; no stronger attacks tested
Quantities used outside the DP accounting	No	–	Class weights $w_{k}$ (from client label counts), aggregation weights $n_{k}$, the Youden threshold on pooled training predictions, pooled z-score statistics, and learning-rate selection on training-derived validation data
Gradient inversion on exposed updates	Partly	Mitigated only by effective noise or secure aggregation	Not tested; not prevented at the ε reported here
Malicious or Byzantine clients (poisoning, backdoors)	No	–	Not covered
Collusion between server and clients	No	–	Not covered
Active attacks on the protocol, client dropout	No	–	Simulation assumes all 13 clients present and honest
Whole-site (client-level) privacy	No	–	Guarantee is record-level, one record per participant
Side channels, timing, and hardware attacks	No	–	Not covered

**Table 7:** Configuration of the ten conventional classifiers.

Classifier	Library	Principal Hyperparameters	Class-Imbalance Handling
Logistic regression	scikit-learn 1.8.0	L2 penalty, C=1.0, 2000 iterations	class weight balanced
RBF-SVM	scikit-learn 1.8.0	C=10, γ= scale; probabilities by 3-fold sigmoid calibration	class weight balanced
Random forest (RF)	scikit-learn 1.8.0	500 trees, defaults otherwise	class weight balanced
Extra trees	scikit-learn 1.8.0	500 trees, defaults otherwise	class weight balanced
HistGB	scikit-learn 1.8.0	500 iterations, defaults otherwise	balanced (augmented); $\left\{0:1,\;1:n^{-}/n^{+}\right\}$ (real-only)
XGBoost	xgboost 3.4.1	500 trees, log-loss, defaults otherwise	scale_pos_weight=1 (augmented); $n^{-}/n^{+}$ (real-only)
LightGBM	lightgbm 4.7.0	500 trees, defaults otherwise	class weight balanced
CatBoost	catboost 1.2.10	500 iterations, defaults otherwise	class weights [1,1] (augmented); $\left[1,n^{-}/n^{+}\right]$ (real-only)
Balanced random forest (BRF)	imbalanced-learn 0.14.2	500 trees	balanced bootstrap undersampling
EasyEnsemble	imbalanced-learn 0.14.2	20 balanced AdaBoost ensembles, defaults otherwise	balanced undersampling

All classifiers: random seed 42; features z-scored with training statistics; hyperparameters fixed a priori and not tuned (defaults for every unlisted setting); no tuning data or validation split by city; the final model was fitted once on the whole training set; the decision threshold is the Youden statistic on out-of-fold predictions of stratified five-fold cross-validation (stratified by label).

**Table 8:** Hyperparameters and protocol settings of the neural and federated experiments.

Setting	Value
Clients and participation	Thirteen natural clients; all participate in every round
Communication rounds *T*	Thirty (main); 50 in the rounds sensitivity run
Local epochs *E*	Five (non-private Adam arms); 3 (SGD, clipping-only, and private arms)
Batch size	$\min(32,n_{k}-1)$; Poisson sampling with $q_{k}=1/\left\lceil n_{k}/B_{k}\right\rceil$ in private arms
Optimizer, learning rate η	Reported private results: SGD without momentum with $\eta^{*}$ chosen on inner validation ($\eta^{*}=0.2$ ResMLP, 0.5 logistic regression; Section 4.5); $\eta =5\times 10^{-3}$ (first submission) shown for comparison. Non-private arms: Adam or SGD with the learning rate chosen on the same inner-validation protocol (Table 17); Adam with $10^{-3}$ (first submission) shown for comparison
Clipping norm *C*; noise multiplier σ	C=1.0; σ∈{2.0,1.2,0.8,0.5} (clipping-only: σ=0)
Privacy parameter δ; accountant	$10^{-5}$; Opacus RDP accountant, one persistent accountant per client
Proximal weight μ	0.01 (0 for FedAvg); 0 and 0.1 in the sensitivity analysis
Focal exponent γ; class weight	γ=2; $w_{k}=n_{k}^{-}/n_{k}^{+}$ computed on each client
Local-only models	Adam, $10^{-3}$, weight decay $10^{-4}$, 200 epochs, batch $\min(32,n_{k}-1)$; threshold on own training data
Centralized ResMLP	Adam, $10^{-3}$, weight decay $10^{-4}$, batch 128, 200 epochs; γ=2 (focal) or none (BCE)
Initialization; seeds	PyTorch defaults; main arms seeds 42–46, sensitivity and client-count seeds 42–44
Stopping and model selection	None: final-round model; no early stopping; test set never used for selection
Decision threshold	Youden statistic on pooled training predictions of the final model (neural) or out-of-fold predictions (conventional)

**Table 9:** Dataset and natural non-IID client distribution across 13 Sialon-II cities. The “Real” columns report the undersampled real participant component of the final balanced training set (Equation (10): 1427 real participants, 248 positive and 1179 negative). This is not the same as the original 2358-participant real training partition. Aug.Prev%, Real Prev%, and Test Prev% are the HIV-positive prevalence within three distinct city-specific populations (augmented training set, undersampled real subset, and unseen test partition); they are not components of a common percentage distribution, and must not be summed or pooled across columns.

City	Aug.N	Aug.Pos	Synthetic	Aug.Neg	Aug.Prev%	Real N	Real Pos	Real Neg	Real Prev%	Test N	Test Pos	Test Neg	Test Prev%
Barcelona	242	147	116	95	60.7	126	31	95	24.6	200	31	169	15.5
Bratislava	153	47	37	106	30.7	116	10	106	8.6	200	10	190	5.0
Brighton	255	162	128	93	63.5	127	34	93	26.8	201	34	167	16.9
Brussels	202	109	86	93	54.0	116	23	93	19.8	190	24	166	12.6
Bucharest	127	86	68	41	67.7	59	18	41	30.5	91	18	73	19.8
Hamburg	214	119	94	95	55.6	120	25	95	20.8	195	25	170	12.8
Lisbon	246	162	128	84	65.9	118	34	84	28.8	186	35	151	18.8
Ljubljana	131	38	30	93	29.0	101	8	93	7.9	173	8	165	4.6
Sofia	139	43	34	96	30.9	105	9	96	8.6	180	8	172	4.4
Stockholm	137	43	34	94	31.4	103	9	94	8.7	178	9	169	5.1
Verona	179	76	60	103	42.5	119	16	103	13.4	200	16	184	8.0
Vilnius	107	19	15	88	17.8	92	4	88	4.3	161	4	157	2.5
Warsaw	226	128	101	98	56.6	125	27	98	21.6	203	27	176	13.3

**Table 10:** Quantitative between-client shift of the 13 natural clients.

City	$n_{k}$	HIV+ (%)	Label JSD	|SMD|	MMD^2^	C2ST AUROC
Barcelona	126	24.6	0.075	0.189	0.0161	0.774
Bratislava	116	8.6	0.112	0.216	0.0235	0.810
Brighton	127	26.8	0.096	0.157	0.0101	0.709
Brussels	116	19.8	0.027	0.199	0.0190	0.764
Bucharest	59	30.5	0.131	0.359	0.0657	0.868
Hamburg	120	20.8	0.037	0.160	0.0123	0.751
Lisbon	118	28.8	0.116	0.201	0.0195	0.785
Ljubljana	101	7.9	0.122	0.148	0.0083	0.708
Sofia	105	8.6	0.112	0.294	0.0384	0.922
Stockholm	103	8.7	0.110	0.249	0.0274	0.789
Verona	119	13.4	0.046	0.185	0.0219	0.806
Vilnius	92	4.3	0.183	0.265	0.0424	0.889
Warsaw	125	21.6	0.045	0.180	0.0174	0.847
Mean			0.093	0.216	0.0248	0.802

Each city is compared with the rest of the federation using its undersampled real training participants. JSD: Jensen–Shannon distance between the city’s label distribution and the pooled one; |SMD|: mean absolute standardized mean difference over the 51 input columns; MMD^2^: unbiased maximum mean discrepancy (Gaussian kernel, median bandwidth); C2ST: cross-validated AUROC of a classifier that separates the city from the rest (0.5 = indistinguishable).

**Table 11:** Conventional machine learning performance on the unseen test set (augmented versus real-only training; identical model order in both blocks).

Model	Training Set	Accuracy	Bal. acc.	Sensitivity	Specificity	MCC	AUROC	AUPRC	Brier	ECE
LR	Augmented ^†^	0.6760	0.5869	0.4739	0.6999	0.1146	0.6288	0.1613	0.2064	0.2776
RBF-SVM	Augmented ^†^	0.7829	0.5297	0.2088	0.8506	0.0504	0.5906	0.1452	0.1781	0.2033
RF	Augmented ^†^	0.8138	0.5382	0.1888	0.8876	0.0721	0.6551	0.1655	0.1457	0.1818
ExtraTrees	Augmented ^†^	0.8003	0.5271	0.1807	0.8734	0.0491	0.6225	0.1459	0.1652	0.1815
HistGB	Augmented ^†^	0.8435	0.5371	0.1486	0.9256	0.0829	0.6220	0.1701	0.1533	0.1497
XGBoost	Augmented ^†^	0.8465	0.5440	0.1606	0.9275	0.0988	0.6132	0.1661	0.1543	0.1520
LightGBM	Augmented ^†^	0.8533	0.5337	0.1285	0.9388	0.0821	0.6128	0.1562	0.1527	0.1539
CatBoost	Augmented ^†^	0.8232	0.5611	0.2289	0.8933	0.1158	0.6510	0.1768	0.1342	0.1376
BRF	Augmented ^†^	0.8367	0.5351	0.1526	0.9175	0.0753	0.6568	0.1693	0.1454	0.1824
EasyEnsemble	Augmented ^†^	0.7405	0.6017	0.4257	0.7776	0.1455	0.6588	0.1864	0.2179	0.3551
LR	Real-only	0.5594	0.6421	0.7470	0.5372	0.1747	0.6919	0.2134	0.2158	0.3386
RBF-SVM	Real-only	0.5305	0.5923	0.6707	0.5140	0.1135	0.6079	0.1520	0.0986	0.0685
RF	Real-only	0.6421	0.6299	0.6145	0.6453	0.1643	0.6741	0.1760	0.1024	0.0687
ExtraTrees	Real-only	0.6344	0.6026	0.5622	0.6430	0.1300	0.6378	0.1561	0.1123	0.0830
HistGB	Real-only	0.4254	0.5991	0.8193	0.3789	0.1270	0.6322	0.1658	0.1539	0.1514
XGBoost	Real-only	0.4584	0.5839	0.7430	0.4248	0.1050	0.6205	0.1530	0.1641	0.1592
LightGBM	Real-only	0.5115	0.5958	0.7028	0.4889	0.1180	0.6341	0.1659	0.1500	0.1412
CatBoost	Real-only	0.6378	0.6346	0.6305	0.6387	0.1696	0.6619	0.1826	0.1622	0.2139
BRF	Real-only	0.5365	0.6328	0.7550	0.5107	0.1634	0.6907	0.1880	0.1789	0.2779
EasyEnsemble	Real-only	0.6035	0.6296	0.6627	0.5965	0.1608	0.6956	0.2355	0.2262	0.3683

Threshold-dependent columns (accuracy, balanced accuracy, sensitivity, specificity, MCC) use the Youden threshold on out-of-fold training predictions. AUROC, AUPRC, Brier score, and ECE do not depend on the threshold. ^†^ In the augmented block, the out-of-fold thresholds are unreliable because synthetic neighbors of held-out real participants remain in the training folds; thus, its threshold-dependent columns (accuracy, balanced accuracy, sensitivity, specificity, MCC) should not be considered and only AUROC, AUPRC, Brier score, and ECE are comparable across blocks. Test prevalence is 10.6% (249 of 2358).

**Table 12:** Dependence of the conventional models on the data partition: repeated stratified 5 × 2 validation.

Model	AUROC	AUPRC	Sensitivity	Specificity	MCC	Original Split: AUROC/AUPRC
Logistic regression	0.664 ± 0.012	0.237 ± 0.019	0.593 ± 0.103	0.639 ± 0.100	0.164 ± 0.020	0.692/0.213
EasyEnsemble	0.696 ± 0.014	0.281 ± 0.024	0.653 ± 0.090	0.610 ± 0.094	0.183 ± 0.021	0.696/0.235

Mean ± SD over ten train/test partitions (five repetitions of two-fold cross-validation stratified by city and label) of the 3785 real participants available in the processed files; training halves have 1892–1893 participants. The pooled prevalence is 13.1%, higher than the 10.6% of the original test partition, so AUPRC is not comparable between columns; AUROC, sensitivity, and specificity are prevalence-invariant.

**Table 13:** Class imbalance handling and augmentation ablation (centralized training).

Model	AUROC	AUPRC	MCC	Sensitivity	Specificity
Centralized ResMLP, real-only, focal loss	0.601 ± 0.006	0.154 ± 0.011	0.076 ± 0.025	0.198 ± 0.035	0.884 ± 0.011
Centralized ResMLP, real-only, weighted BCE	0.605 ± 0.013	0.153 ± 0.009	0.083 ± 0.016	0.222 ± 0.034	0.871 ± 0.017
Centralized ResMLP, augmented, focal loss	0.577 ± 0.008	0.140 ± 0.006	0.075 ± 0.015	0.320 ± 0.026	0.783 ± 0.017
Centralized ResMLP, augmented, weighted BCE	0.582 ± 0.014	0.138 ± 0.005	0.088 ± 0.020	0.341 ± 0.031	0.780 ± 0.011
Centralized ResMLP, real-only, focal loss, validation-tuned Adam η=0.01	0.591 ± 0.006	0.163 ± 0.009	0.090 ± 0.014	0.263 ± 0.024	0.845 ± 0.031
Logistic regression, augmented, class weights (from Table 11)	0.6288	0.1613	0.1146	0.4739	0.6999
Logistic regression, real-only, class weights (from Table 11)	0.6919	0.2134	0.1747	0.7470	0.5372
RBF-SVM, augmented, class weights (from Table 11)	0.5906	0.1452	0.0504	0.2088	0.8506
RBF-SVM, real-only, class weights (from Table 11)	0.6079	0.1520	0.1135	0.6707	0.5140
Random forest, augmented, class weights (from Table 11)	0.6551	0.1655	0.0721	0.1888	0.8876
Random forest, real-only, class weights (from Table 11)	0.6741	0.1760	0.1643	0.6145	0.6453

Neural rows: mean ± SD over five seeds, same GroupNorm ResMLP as the federated arms. Conventional rows: single fixed seed, rows copied from Table 11 and labeled with their training set.

**Table 14:** Federated training on real-only versus city-wise augmented data (non-private).

Arm	AUROC	AUPRC	Sensitivity	Specificity
FedAvg, real-only	0.607 ± 0.009	0.157 ± 0.006	0.378 ± 0.019	0.768 ± 0.018
FedAvg, augmented (per-city SMOTENC)	0.598 ± 0.016	0.154 ± 0.005	0.416 ± 0.035	0.726 ± 0.034
FedProx, real-only	0.607 ± 0.011	0.158 ± 0.007	0.381 ± 0.040	0.771 ± 0.016
FedProx, augmented (per-city SMOTENC)	0.597 ± 0.016	0.154 ± 0.007	0.389 ± 0.025	0.744 ± 0.022

Non-private arms, 13 clients, mean ± SD over five seeds. Paired AUROC differences (augmented minus real-only): FedProx, non-DP: −0.011 (95% CI −0.027, +0.008); FedAvg, non-DP: −0.009 (95% CI −0.027, +0.009).

**Table 15:** Federated neural results with the ResMLP-DP classifier: non-private baselines, clipping-only, and HIV-PrivFed.

Model	ε	AUROC	AUPRC	MCC	Sensitivity	Specificity	MIA AUROC
Local-only (mean of 13 local models)	–	0.565 ± 0.005	0.132 ± 0.002	0.019 ± 0.003	0.045 ± 0.003	0.968 ± 0.002	–
FedAvg (Adam η=0.001, E=5)	–	0.607 ± 0.009	0.157 ± 0.006	0.104 ± 0.021	0.378 ± 0.019	0.768 ± 0.018	0.559 ± 0.011
FedProx (Adam η=0.001, E=5)	–	0.607 ± 0.011	0.158 ± 0.007	0.108 ± 0.024	0.381 ± 0.040	0.771 ± 0.016	0.559 ± 0.009
FedAvg (Adam, E=5), validation-tuned η=0.0003	–	0.628 ± 0.007	0.164 ± 0.007	0.116 ± 0.005	0.481 ± 0.026	0.696 ± 0.020	0.547 ± 0.009
FedProx (Adam, E=5), validation-tuned η=0.0003	–	0.628 ± 0.007	0.164 ± 0.007	0.117 ± 0.007	0.486 ± 0.031	0.693 ± 0.023	0.547 ± 0.009
FedProx, SGD E=3 (no clipping, no noise), validation-tuned η=0.1	–	0.622 ± 0.012	0.155 ± 0.010	0.095 ± 0.004	0.367 ± 0.042	0.766 ± 0.037	0.570 ± 0.014
FedProx, SGD η=0.005, E=3 (no clipping, no noise)	–	0.587 ± 0.016	0.136 ± 0.005	0.079 ± 0.018	0.579 ± 0.065	0.549 ± 0.048	0.521 ± 0.009
FedProx, SGD $\eta^{*}=0.2$, E=3 (no clipping, no noise)	–	0.622 ± 0.010	0.160 ± 0.005	0.104 ± 0.025	0.417 ± 0.049	0.735 ± 0.023	0.581 ± 0.012
Clipping only (σ=0), η=0.005	–	0.534 ± 0.036	0.120 ± 0.016	0.025 ± 0.019	0.499 ± 0.226	0.540 ± 0.248	0.477 ± 0.008
HIV-PrivFed σ=2.0, η=0.005	22.1	0.533 ± 0.037	0.119 ± 0.016	0.023 ± 0.029	0.489 ± 0.244	0.547 ± 0.268	0.478 ± 0.008
HIV-PrivFed σ=1.2, η=0.005	47.2	0.533 ± 0.036	0.120 ± 0.016	0.033 ± 0.014	0.504 ± 0.226	0.545 ± 0.228	0.477 ± 0.008
HIV-PrivFed σ=0.8, η=0.005	92.9	0.534 ± 0.036	0.120 ± 0.016	0.031 ± 0.019	0.453 ± 0.184	0.594 ± 0.185	0.477 ± 0.008
HIV-PrivFed σ=0.5, η=0.005	224.1	0.534 ± 0.036	0.120 ± 0.016	0.034 ± 0.011	0.490 ± 0.215	0.560 ± 0.214	0.477 ± 0.008
Clipping only (σ=0), $\eta^{*}=0.2$	–	0.651 ± 0.010	0.176 ± 0.005	0.138 ± 0.013	0.500 ± 0.082	0.708 ± 0.059	0.490 ± 0.003
HIV-PrivFed σ=2.0, $\eta^{*}=0.2$	22.1	0.629 ± 0.015	0.166 ± 0.009	0.122 ± 0.012	0.519 ± 0.102	0.667 ± 0.094	0.472 ± 0.008
HIV-PrivFed σ=1.2, $\eta^{*}=0.2$	47.2	0.647 ± 0.015	0.175 ± 0.007	0.140 ± 0.012	0.555 ± 0.026	0.662 ± 0.031	0.480 ± 0.005
HIV-PrivFed σ=0.8, $\eta^{*}=0.2$	92.9	0.650 ± 0.015	0.177 ± 0.008	0.133 ± 0.015	0.569 ± 0.056	0.641 ± 0.038	0.485 ± 0.003
HIV-PrivFed σ=0.5, $\eta^{*}=0.2$	224.1	0.650 ± 0.014	0.177 ± 0.009	0.127 ± 0.009	0.528 ± 0.058	0.669 ± 0.048	0.488 ± 0.003
HIV-PrivFed calibrated to ε=8 (σ=4.4), $\eta^{*}=0.2$	8.0	0.553 ± 0.023	0.129 ± 0.010	0.046 ± 0.017	0.443 ± 0.266	0.620 ± 0.261	0.466 ± 0.005
HIV-PrivFed calibrated to ε=4 (σ=7.9), $\eta^{*}=0.2$	4.0	0.524 ± 0.025	0.115 ± 0.004	0.034 ± 0.026	0.490 ± 0.345	0.557 ± 0.319	0.465 ± 0.008
HIV-PrivFed calibrated to ε=2 (σ=14.5), $\eta^{*}=0.2$	2.0	0.515 ± 0.015	0.113 ± 0.004	0.019 ± 0.010	0.451 ± 0.235	0.575 ± 0.232	0.467 ± 0.007
HIV-PrivFed calibrated to ε=1 (σ=27.3), $\eta^{*}=0.2$	1.0	0.523 ± 0.012	0.116 ± 0.004	0.024 ± 0.015	0.503 ± 0.276	0.529 ± 0.277	0.471 ± 0.006

Mean ± SD over five independent runs (seeds 42–46) on the unseen test set; every row uses the same 13 real clients, 30 rounds, and decision rule. ε: worst-client budget from the persistent RDP accountants at $\delta =10^{-5}$ (Table 5); MIA: AUROC of the loss threshold membership inference audit (0.5 = no detectable leakage; a weak attack, not a privacy proof). $\eta^{*}$ is the learning rate chosen on inner validation data (Table 20).

**Table 16:** Paired statistical comparisons (AUROC, unseen test set).

Comparison (A Versus B)	A	B	A − B	95% CI	Bootstrap *p*	Holm *p*	Wilcoxon *p*
FedProx vs. local-only ^†^	0.607	0.565	+0.042	(+0.017, +0.065)	<0.001	–	0.062
noise effect, sigma 0.8, tuned lr ^†^	0.651	0.650	+0.001	(−0.007, +0.008)	0.854	–	1.000
noise effect, worst-client epsilon 8 ^†^	0.651	0.553	+0.098	(+0.070, +0.127)	<0.001	–	0.062
HIV-PrivFed (sigma 0.8, tuned) vs. validation-tuned non-private FedProx ^†^	0.650	0.628	+0.022	(+0.005, +0.039)	0.010	–	0.125
FedAvg vs. local-only	0.607	0.565	+0.042	(+0.016, +0.065)	<0.001	0.029	0.062
FedProx vs. FedAvg	0.607	0.607	+0.000	(−0.001, +0.002)	0.594	1.000	0.812
Adam (E = 5) vs. SGD 0.005 (E = 3), non-private	0.607	0.587	+0.021	(−0.005, +0.046)	0.126	1.000	0.062
tuned lr vs. lr 0.005, non-private SGD	0.622	0.587	+0.036	(+0.010, +0.059)	0.008	0.104	0.062
clipping effect at lr 0.005	0.587	0.534	+0.053	(+0.037, +0.068)	<0.001	0.029	0.125
clipping effect at tuned lr	0.622	0.651	−0.028	(−0.047, −0.009)	0.010	0.120	0.062
noise effect, sigma 0.8, lr 0.005	0.534	0.534	+0.000	(−0.000, +0.001)	0.510	1.000	0.625
noise effect, sigma 0.5, tuned lr	0.651	0.650	+0.000	(−0.005, +0.005)	0.896	1.000	0.812
sigma 2.0 vs. 1.2 (tuned lr)	0.629	0.647	−0.019	(−0.027, −0.010)	<0.001	0.029	0.062
sigma 1.2 vs. 0.8 (tuned lr)	0.647	0.650	−0.003	(−0.007, +0.002)	0.282	1.000	0.312
sigma 0.8 vs. 0.5 (tuned lr)	0.650	0.650	−0.000	(−0.004, +0.003)	0.916	1.000	1.000
sigma 2.0 vs. 0.5 (tuned lr)	0.629	0.650	−0.022	(−0.036, −0.007)	<0.001	0.029	0.062
HIV-PrivFed (sigma 0.8, tuned) vs. non-private FedProx	0.650	0.607	+0.043	(+0.020, +0.064)	<0.001	0.029	0.062
noise effect, worst-client epsilon 4	0.651	0.524	+0.127	(+0.097, +0.158)	<0.001	0.029	0.062
noise effect, worst-client epsilon 1	0.651	0.523	+0.128	(+0.098, +0.159)	<0.001	0.029	0.062
HIV-PrivFed (epsilon 8) vs. local-only	0.553	0.565	−0.012	(−0.031, +0.006)	0.188	1.000	0.438
HIV-PrivFed (sigma 0.8, tuned) vs. local-only	0.650	0.565	+0.085	(+0.065, +0.106)	<0.001	0.029	0.062
federated LR vs. federated ResMLP	0.647	0.607	+0.040	(+0.015, +0.064)	0.004	0.064	0.062
FedProx-LR vs. local-only LR	0.647	0.560	+0.087	(+0.064, +0.112)	<0.001	0.029	0.062
DP-LR vs. DP-ResMLP (sigma 0.8, tuned lr)	0.663	0.650	+0.013	(−0.009, +0.036)	0.224	1.000	0.312
LR noise effect (sigma 0.8, tuned lr)	0.663	0.672	−0.009	(−0.015, −0.004)	<0.001	0.029	0.312
augmentation effect (FedProx, non-DP)	0.597	0.607	−0.011	(−0.027, +0.008)	0.230	1.000	0.125
augmentation effect (FedAvg, non-DP)	0.598	0.607	−0.009	(−0.027, +0.009)	0.310	1.000	0.125
validation-tuned FedProx vs. local-only	0.628	0.565	+0.063	(+0.040, +0.085)	<0.001	0.029	0.062
validation-tuned vs. untuned Adam FedProx	0.628	0.607	+0.020	(+0.008, +0.033)	0.002	0.034	0.062
FedProx vs. FedAvg (validation-tuned)	0.628	0.628	+0.000	(−0.000, +0.000)	0.660	1.000	0.625
HIV-PrivFed (sigma 0.8, tuned) vs. validation-tuned non-private SGD	0.650	0.622	+0.028	(+0.009, +0.049)	0.006	0.084	0.125
HIV-PrivFed (epsilon 8) vs. validation-tuned non-private FedProx	0.553	0.628	−0.075	(−0.104, −0.047)	<0.001	0.029	0.062
clipping effect at the validation-tuned non-private rate	0.622	0.651	−0.029	(−0.046, −0.010)	0.004	0.064	0.062

AUROC of A and B averaged over five seeds; difference A − B; 95% percentile interval and two-sided *p* from 1000 paired stratified bootstrap resamples of the test participants (the same resamples for A and B; differences are averaged over the five seeds inside every resample). ^†^ Primary comparisons (designated before the final analysis), reported with raw bootstrap *p*; all other rows are exploratory, and their Holm-adjusted *p* is computed over the 29 exploratory comparisons. Bootstrap *p* cannot be smaller than 1/1000, and is shown as <0.001 at that floor. Wilcoxon signed-rank *p* over the five paired seeds (smallest attainable value 0.0625) is shown for information only. In the rows named “effect”, A is the model without the studied factor (unclipped SGD for clipping effects, clipping-only for noise effects, real-only for augmentation effects), so a negative A − B means that the factor raised the AUROC.

**Table 17:** Learning rate selection for the non-private arms on inner validation data.

Arm	Learning Rate	Validation AUROC
FedProx, SGD (E=3)	0.005	0.557 ± 0.072
	0.02	0.603 ± 0.054
	0.05	0.610 ± 0.033
	**0.1**	**0.612 ± 0.031**
	0.2	0.610 ± 0.033
	0.5	0.607 ± 0.037
FedAvg, Adam (E=5)	0.0001	0.605 ± 0.057
	**0.0003**	**0.628 ± 0.039**
	0.001	0.611 ± 0.027
	0.003	0.600 ± 0.043
	0.01	0.593 ± 0.030
FedProx, Adam (E=5)	0.0001	0.605 ± 0.057
	**0.0003**	**0.627 ± 0.039**
	0.001	0.609 ± 0.028
	0.003	0.604 ± 0.037
	0.01	0.622 ± 0.020
Centralized ResMLP, Adam	0.0001	0.611 ± 0.040
	0.0003	0.615 ± 0.037
	0.001	0.606 ± 0.040
	0.003	0.593 ± 0.042
	**0.01**	**0.619 ± 0.020**

Validation AUROC (mean ± SD over four validation splits) on the held-out 20% of every client’s real training participants (the centralized model uses the pooled 80%); the test partition is not used. Bold: selected learning rate. The SGD arm uses the grid of Table 20; the Adam arms use a grid from $10^{-4}$ to $10^{-2}$ because Adam learning rates are on a different scale.

**Table 18:** Model-class control, consisting of the same federated protocols with logistic regression (52 parameters).

Model	ε	AUROC	AUPRC	MCC	Sensitivity	Specificity	MIA AUROC
Local-only (mean of 13 local models)	–	0.560 ± 0.002	0.135 ± 0.001	0.053 ± 0.004	0.501 ± 0.012	0.583 ± 0.015	–
FedAvg (Adam η=0.001, E=5)	–	0.647 ± 0.015	0.182 ± 0.007	0.148 ± 0.017	0.514 ± 0.111	0.705 ± 0.096	0.498 ± 0.010
FedProx (Adam η=0.001, E=5)	–	0.647 ± 0.015	0.182 ± 0.007	0.149 ± 0.017	0.514 ± 0.111	0.705 ± 0.096	0.498 ± 0.010
FedProx, SGD η=0.005, E=3 (no clipping, no noise)	–	0.641 ± 0.025	0.192 ± 0.019	0.133 ± 0.027	0.582 ± 0.087	0.626 ± 0.085	0.505 ± 0.017
FedProx, SGD $\eta^{*}=0.5$, E=3 (no clipping, no noise)	–	0.628 ± 0.026	0.171 ± 0.022	0.127 ± 0.026	0.602 ± 0.033	0.600 ± 0.062	0.508 ± 0.007
Clipping only (σ=0), η=0.005	–	0.578 ± 0.023	0.148 ± 0.007	0.070 ± 0.023	0.483 ± 0.148	0.623 ± 0.140	0.480 ± 0.010
Clipping only (σ=0), $\eta^{*}=0.5$	–	0.672 ± 0.003	0.208 ± 0.002	0.178 ± 0.010	0.510 ± 0.046	0.748 ± 0.044	0.464 ± 0.006
HIV-PrivFed σ=2.0, $\eta^{*}=0.5$	22.1	0.631 ± 0.024	0.182 ± 0.025	0.130 ± 0.029	0.517 ± 0.057	0.680 ± 0.070	0.468 ± 0.002
HIV-PrivFed σ=1.2, $\eta^{*}=0.5$	47.2	0.652 ± 0.014	0.192 ± 0.019	0.155 ± 0.024	0.479 ± 0.058	0.745 ± 0.058	0.466 ± 0.002
HIV-PrivFed σ=0.8, $\eta^{*}=0.5$	92.9	0.663 ± 0.009	0.198 ± 0.015	0.160 ± 0.016	0.448 ± 0.081	0.775 ± 0.052	0.465 ± 0.003
HIV-PrivFed σ=0.5, $\eta^{*}=0.5$	224.1	0.668 ± 0.005	0.202 ± 0.012	0.170 ± 0.007	0.522 ± 0.078	0.728 ± 0.063	0.465 ± 0.004

Mean ± SD over five independent runs (seeds 42–46) on the unseen test set; every row uses the same 13 real clients, 30 rounds, and decision rule. ε: worst-client budget from the persistent RDP accountants at $\delta =10^{-5}$ (Table 5); MIA: AUROC of the loss threshold membership inference audit (0.5 = no detectable leakage; a weak attack, not a privacy proof). $\eta^{*}$ is the learning rate chosen on inner validation data (Table 20).

**Table 19:** Sensitivity of the achieved privacy budget ε to cohort size, δ, local epochs, rounds, and batch size (analytic accounting).

Factor	Setting	Worst-Case ε (RDP)
Client cohort size $n_{k}$	$n_{k}=59$ (q=0.500, 180 steps)	92.9
	$n_{k}=100$ (q=0.250, 360 steps)	66.1
	$n_{k}=127$ (q=0.250, 360 steps)	66.1
	$n_{k}=250$ (q=0.125, 720 steps)	45.5
	$n_{k}=500$ (q=0.062, 1440 steps)	30.7
	$n_{k}=1000$ (q=0.031, 2880 steps)	20.4
	$n_{k}=1427$ (q=0.022, 4050 steps)	16.7
	$n_{k}=3000$ (q=0.011, 8460 steps)	10.7
	$n_{k}=10,000$ (q=0.003, 28,170 steps)	5.2
Full real partition (client sizes from the test partition, mean 181.4), worst client	σ=2.0 (q=0.333, 270 steps)	17.4 (undersampled: 22.1)
	σ=1.2 (q=0.333, 270 steps)	37.5 (undersampled: 47.2)
	σ=0.8 (q=0.333, 270 steps)	76.6 (undersampled: 92.9)
	σ=0.5 (q=0.333, 270 steps)	196.8 (undersampled: 224.1)
Failure probability δ (worst client)	$\delta =10^{-6}$	98.7
	$\delta =10^{-5}$	92.9
	$\delta =10^{-4}$	87.2
	$1/N=7.0\times 10^{-4}$	81.1
Local epochs *E* and rounds *T* (worst client)	E=1, T=10	23.4
	E=1, T=30	44.7
	E=1, T=50	62.1
	E=3, T=10	44.7
	E=3, T=30	92.9
	E=3, T=50	134.7
	E=5, T=10	62.1
	E=5, T=30	134.7
	E=5, T=50	200.5
Batch-size parameter *B* (worst client)	B=8 (q=0.125, 720 steps)	45.5
	B=16 (q=0.250, 360 steps)	66.1
	B=32 (q=0.500, 180 steps)	92.9
	B=58 (q=0.500, 180 steps)	92.9
Noise multiplier needed (worst client)	target ε=1	σ=27.3
	target ε=2	σ=14.5
	target ε=4	σ=7.9
	target ε=8	σ=4.4
	target ε=10	σ=3.7

Analytic RDP accounting at *σ* = 0.8, *C*-independent, *B* = 32, *E* = 3, *T* = 30, *δ* = 10^−5^ unless stated. The cohort size rows use a single client of that size; *N* = 1,427 is the whole undersampled real subset treated as one client. The last block gives the noise multiplier at which the worst client reaches the target *ε*.

**Table 20:** Learning rate selection for differentially private training on inner validation data.

Learning Rate η	ResMLP-DP: Validation AUROC	Logistic Regression: Validation AUROC
0.005	0.538 ± 0.063	0.541 ± 0.014
0.02	0.568 ± 0.057	0.589 ± 0.034
0.05	0.597 ± 0.059	0.606 ± 0.028
0.1	0.613 ± 0.055	0.608 ± 0.029
0.2	**0.623 ± 0.042**	0.611 ± 0.031
0.5	0.606 ± 0.027	**0.617 ± 0.031**

Validation AUROC (mean ± SD over four validation splits) of HIV-PrivFed at σ=0.8 (ε unchanged by η) trained on 80% of every client’s real training participants and evaluated on the held-out 20%; the test partition is not used. Bold: selected $\eta^{*}$. Validation sets are small (about 285 participants, about 50 positives), so differences below about 0.03 are within noise. Selection on training-derived data is not covered by the (ε,δ) accounting.

**Table 21:** Sensitivity of differentially private utility and privacy budget to hyperparameters.

Factor	Setting	AUROC	AUPRC	Worst-Client ε
Reference	C=1, E=3, T=30, μ=0.01, $\eta^{*}=0.2$	0.648 ± 0.021	0.178 ± 0.011	92.9
Clipping norm *C*	0.5	0.641 ± 0.023	0.174 ± 0.013	92.9
	2.0	0.650 ± 0.020	0.177 ± 0.007	92.9
Local epochs *E*	1	0.642 ± 0.021	0.168 ± 0.018	44.7
	5	0.632 ± 0.009	0.174 ± 0.007	134.7
Proximal weight μ	0 (no proximal term)	0.648 ± 0.020	0.178 ± 0.011	92.9
	0.1	0.648 ± 0.020	0.179 ± 0.011	92.9
Rounds *T*	10	0.623 ± 0.027	0.159 ± 0.011	44.7
	20	0.643 ± 0.018	0.180 ± 0.007	70.3
	30	0.648 ± 0.021	0.178 ± 0.011	92.9
	40	0.641 ± 0.017	0.180 ± 0.004	115.0
	50	0.650 ± 0.021	0.186 ± 0.002	134.7

One-factor-at-a-time changes around the reference configuration (HIV-PrivFed, σ=0.8); mean ± SD over three seeds (42–44). The privacy budget does not depend at all on *C* and μ (it depends on σ, $q_{k}$, and the number of steps), and depends on *E* and *T* through the number of steps. Rounds: AUROC/AUPRC and ε are for that round of a single 50-round run. The learning rate is covered in Table 20 and the noise multiplier in Table 15.

**Table 22:** Effects of number of clients and natural heterogeneity (non-private FedProx and HIV-PrivFed at σ=0.8).

*K*	Client Construction	Client Sizes	Label JSD	FedProx AUROC	HIV-PrivFed AUROC	Worst-Client ε
2	Natural cities, sorted by prevalence	675–752	0.082	0.577 ± 0.012	0.611 ± 0.012	25.5
2	Natural cities, random groups	619–808	0.012	0.581 ± 0.006	–	–
2	IID clients	713–714	0.000	0.599 ± 0.003	0.628 ± 0.015	24.8
3	Natural cities, sorted by prevalence	430–517	0.081	0.590 ± 0.011	0.638 ± 0.008	33.2
3	Natural cities, random groups	372–574	0.055	0.590 ± 0.016	–	–
3	IID clients	475–476	0.001	0.592 ± 0.004	0.644 ± 0.014	31.9
5	Natural cities, sorted by prevalence	177–361	0.093	0.576 ± 0.003	0.649 ± 0.007	53.4
5	Natural cities, random groups	151–365	0.056	0.591 ± 0.015	–	–
5	IID clients	284–286	0.001	0.597 ± 0.003	0.654 ± 0.007	42.7
13	Natural cities (the 13 clients)	59–127	0.093	0.601 ± 0.010	0.648 ± 0.021	92.9
13	IID clients	109–111	0.001	0.613 ± 0.004	0.648 ± 0.010	66.1

Same 1427 real training participants, protocol, and test set in every row; mean ± SD over three seeds. Heterogeneity: mean Jensen–Shannon distance between each client’s label distribution and the pooled one (seed 42 grouping). IID clients are random label-stratified partitions of the same participants. Private runs: σ=0.8, batch parameter 32 for every *K*, learning rate $\eta^{*}$.

**Table 23:** Client-level performance with 95% bootstrap intervals: FedAvg (non-private).

City	Test pos.	Sensitivity (95% CI)	Specificity (95% CI)	AUROC (95% CI)	AUPRC (95% CI)
Barcelona	31	0.31 (0.21, 0.41)	0.75 (0.70, 0.79)	0.50 (0.40, 0.58)	0.19 (0.15, 0.27)
Bratislava	10	0.18 (0.06, 0.34)	0.82 (0.78, 0.86)	0.53 (0.42, 0.65)	0.07 (0.05, 0.15)
Brighton	34	0.54 (0.42, 0.66)	0.70 (0.64, 0.75)	0.64 (0.57, 0.72)	0.24 (0.21, 0.33)
Brussels	24	0.49 (0.33, 0.66)	0.72 (0.67, 0.77)	0.67 (0.57, 0.76)	0.27 (0.19, 0.41)
Bucharest	18	0.26 (0.11, 0.42)	0.87 (0.81, 0.93)	0.55 (0.42, 0.68)	0.28 (0.20, 0.45)
Hamburg	25	0.43 (0.30, 0.58)	0.65 (0.60, 0.71)	0.58 (0.50, 0.66)	0.16 (0.14, 0.23)
Lisbon	35	0.30 (0.19, 0.42)	0.80 (0.75, 0.85)	0.59 (0.51, 0.67)	0.27 (0.21, 0.38)
Ljubljana ^†^	8	0.30 (0.00, 0.62)	0.79 (0.74, 0.83)	0.55 (0.36, 0.74)	0.12 (0.05, 0.36)
Sofia ^†^	8	0.07 (0.00, 0.17)	0.79 (0.74, 0.83)	0.47 (0.37, 0.59)	0.05 (0.04, 0.08)
Stockholm ^†^	9	0.53 (0.33, 0.73)	0.66 (0.61, 0.72)	0.63 (0.51, 0.75)	0.08 (0.06, 0.18)
Verona	16	0.47 (0.30, 0.64)	0.87 (0.83, 0.90)	0.78 (0.65, 0.87)	0.42 (0.25, 0.61)
Vilnius ^†^	4	0.25 (0.05, 0.50)	0.83 (0.78, 0.87)	0.59 (0.36, 0.81)	0.10 (0.03, 0.25)
Warsaw	27	0.37 (0.24, 0.50)	0.78 (0.74, 0.82)	0.61 (0.51, 0.70)	0.24 (0.18, 0.36)
Gap (max − min), all 13 cities		0.47	0.22	0.31	0.37
Gap (max − min), 9 stable cities		0.36	0.22	0.28	0.35

Point estimates are the mean over five seeds at the global Youden threshold; intervals are 95% percentile bootstrap intervals within each city (1000 resamples preserving the number of positives). ^†^ Fewer than ten HIV-positive test participants, hence statistically unstable and shown for transparency only. The test AUPRC baseline of each city is its prevalence (Table 9).

**Table 24:** Client-level performance with 95% bootstrap intervals: HIV-PrivFed (σ=0.8, tuned learning rate).

City	Test pos.	Sensitivity (95% CI)	Specificity (95% CI)	AUROC (95% CI)	AUPRC (95% CI)
Barcelona	31	0.55 (0.42, 0.68)	0.57 (0.52, 0.62)	0.58 (0.47, 0.67)	0.22 (0.17, 0.32)
Bratislava	10	0.42 (0.22, 0.62)	0.75 (0.71, 0.80)	0.62 (0.49, 0.74)	0.10 (0.06, 0.23)
Brighton	34	0.74 (0.64, 0.82)	0.53 (0.47, 0.59)	0.67 (0.60, 0.74)	0.27 (0.23, 0.37)
Brussels	24	0.78 (0.64, 0.90)	0.49 (0.43, 0.54)	0.70 (0.60, 0.79)	0.29 (0.20, 0.44)
Bucharest	18	0.31 (0.17, 0.47)	0.76 (0.69, 0.83)	0.59 (0.49, 0.70)	0.29 (0.22, 0.45)
Hamburg	25	0.62 (0.50, 0.76)	0.53 (0.47, 0.59)	0.61 (0.51, 0.71)	0.21 (0.16, 0.32)
Lisbon	35	0.43 (0.31, 0.55)	0.72 (0.66, 0.77)	0.64 (0.55, 0.71)	0.30 (0.24, 0.41)
Ljubljana ^†^	8	0.45 (0.20, 0.72)	0.64 (0.59, 0.70)	0.60 (0.44, 0.76)	0.10 (0.05, 0.28)
Sofia ^†^	8	0.40 (0.28, 0.53)	0.69 (0.64, 0.75)	0.58 (0.50, 0.66)	0.06 (0.05, 0.09)
Stockholm ^†^	9	0.64 (0.40, 0.84)	0.51 (0.45, 0.57)	0.61 (0.46, 0.75)	0.10 (0.06, 0.23)
Verona	16	0.64 (0.44, 0.81)	0.78 (0.74, 0.82)	0.78 (0.66, 0.88)	0.39 (0.23, 0.62)
Vilnius ^†^	4	0.30 (0.05, 0.65)	0.73 (0.68, 0.78)	0.65 (0.51, 0.79)	0.07 (0.04, 0.22)
Warsaw	27	0.60 (0.45, 0.73)	0.67 (0.61, 0.72)	0.68 (0.59, 0.76)	0.21 (0.17, 0.30)
Gap (max − min), all 13 cities		0.48	0.30	0.20	0.33
Gap (max − min), 9 stable cities		0.47	0.30	0.20	0.28

Point estimates are the mean over five seeds at the global Youden threshold; intervals are 95% percentile bootstrap intervals within each city (1000 resamples preserving the number of positives). ^†^ Fewer than ten HIV-positive test participants, hence statistically unstable and shown for transparency only. The test AUPRC baseline of each city is its prevalence (Table 9).

**Table 25:** Global versus city-specific decision thresholds: FedAvg (non-private).

City	Test pos.	Sens., Global $\tau^{*}$	FPR, Global $\tau^{*}$	Sens., City $\tau_{k}$	FPR, City $\tau_{k}$
Barcelona	31	0.31	0.25	0.22	0.16
Bratislava	10	0.18	0.18	0.18	0.20
Brighton	34	0.54	0.30	0.45	0.25
Brussels	24	0.49	0.28	0.49	0.23
Bucharest	18	0.26	0.13	0.40	0.29
Hamburg	25	0.43	0.35	0.37	0.31
Lisbon	35	0.30	0.20	0.50	0.38
Ljubljana ^†^	8	0.30	0.21	0.20	0.05
Sofia ^†^	8	0.07	0.21	0.03	0.14
Stockholm ^†^	9	0.53	0.34	0.29	0.27
Verona	16	0.47	0.13	0.53	0.16
Vilnius ^†^	4	0.25	0.17	0.15	0.06
Warsaw	27	0.37	0.22	0.34	0.20
Gap (max − min), all cities		0.47	0.22	0.50	0.33
Gap, stable cities		0.36	0.22	0.35	0.22

Mean over five seeds. City-specific thresholds are Youden thresholds computed from the global model’s predictions on each city’s own training participants. ^†^ Fewer than ten positive test participants.

**Table 26:** Global versus city-specific decision thresholds: HIV-PrivFed (σ=0.8, tuned learning rate).

City	Test pos.	Sens., Global $\tau^{*}$	FPR, Global $\tau^{*}$	Sens., City $\tau_{k}$	FPR, City $\tau_{k}$
Barcelona	31	0.55	0.43	0.35	0.23
Bratislava	10	0.42	0.25	0.42	0.29
Brighton	34	0.74	0.47	0.49	0.30
Brussels	24	0.78	0.51	0.67	0.41
Bucharest	18	0.31	0.24	0.50	0.35
Hamburg	25	0.62	0.47	0.54	0.36
Lisbon	35	0.43	0.28	0.49	0.31
Ljubljana ^†^	8	0.45	0.36	0.33	0.22
Sofia ^†^	8	0.40	0.31	0.23	0.27
Stockholm ^†^	9	0.64	0.49	0.47	0.33
Verona	16	0.64	0.22	0.75	0.36
Vilnius ^†^	4	0.30	0.27	0.65	0.43
Warsaw	27	0.60	0.33	0.61	0.33
Gap (max − min), all cities		0.48	0.30	0.53	0.22
Gap, stable cities		0.47	0.30	0.40	0.18

Mean over five seeds. City-specific thresholds are Youden thresholds computed from the global model’s predictions on each city’s own training participants. ^†^ Fewer than ten positive test participants.

**Table 27:** Bootstrap 95% confidence intervals (1000 stratified resamples of the test set; out-of-fold training-derived thresholds).

Model	AUROC (95% CI)	AUPRC (95% CI)	MCC (95% CI)
EasyEnsemble (real-only)	0.6956 (0.6634, 0.7258)	0.2355 (0.2021, 0.2798)	0.1608 (0.1234, 0.1974)
Logistic regression (real-only)	0.6919 (0.6577, 0.7264)	0.2134 (0.1860, 0.2586)	0.1747 (0.1396, 0.2097)
Balanced random forest (real-only)	0.6907 (0.6602, 0.7215)	0.1880 (0.1681, 0.2176)	0.1634 (0.1302, 0.1969)
CatBoost (real-only)	0.6619 (0.6270, 0.6962)	0.1826 (0.1610, 0.2157)	0.1696 (0.1332, 0.2085)

**Table 28:** Paired bootstrap comparison, EasyEnsemble minus logistic regression (real-only training).

Metric	Mean Difference	95% CI	Bootstrap *p*	Excludes 0?
AUROC	0.0038	(−0.0239, 0.0321)	0.806	No
AUPRC	0.0204	(−0.0122, 0.0550)	0.228	No
MCC	−0.0135	(−0.0493, 0.0230)	0.490	No
Sensitivity	−0.0836	(−0.1365, −0.0281)	0.006	Yes
Specificity	0.0592	(0.0379, 0.0801)	0.001	Yes

**Table 29:** Theoretical time and space complexity by model family.

Model	Time	Space
LR	O(n·d)	O(d)
RF/ExtraTrees/BRF	O(n·T·d·logn)	O(T·n)
XGB/LGBM/CatBoost	O(n·d·T)	O(T·d)
EasyEnsemble	$O(K\cdot n_{\mathrm{sub}}\cdot T\cdot d)$	O(K·T·d)
ResMLP (centralized)	$O(\mathrm{epochs}\cdot n\cdot d^{2})$	$O(d^{2})$
FedAvg/FedProx	$O(R\cdot K\cdot n_{\mathrm{avg}}\cdot d^{2})$	$O(K\cdot d^{2})$
HIV-PrivFed (DP-FedProx)	$O\left(R\cdot K\cdot n_{\mathrm{avg}}\cdot d^{2}/B\right)+O\left(K\cdot d\right)\;\left(\mathrm{masking}\right)+O\left(K\right)\;\left(\mathrm{AES}\right)$	$O(K\cdot d^{2}\cdot B)$

**Table 30:** Measured training time and inference latency of the evaluated classifiers.

Model	Training Time (s)	Latency Mean (ms)	Latency P95 (ms)	Latency P99 (ms)	Whole Test Set (ms)
LR	0.01	0.068	0.107	0.135	0.2
RBF-SVM	0.16	1.730	2.235	3.887	246.6
RF	1.16	33.583	40.014	57.303	133.0
ExtraTrees	1.20	33.655	39.122	54.019	164.0
HistGB	1.22	3.576	3.834	4.107	77.4
XGBoost	0.55	0.389	0.724	1.269	30.0
LightGBM	0.69	0.558	0.639	2.654	80.4
CatBoost	1.08	0.213	0.307	0.517	4.0
BRF	1.43	34.683	41.295	76.186	127.3
EasyEnsemble	1.70	82.235	96.794	141.852	234.9
ResMLP-DP (neural)	–	0.080	0.112	0.158	4.6
Logistic regression (neural, 52 parameters)	–	0.014	0.015	0.045	0.2

Single virtual CPU core, one participant per call (1000 calls after warm-up), and one call for the whole test set (2358 participants). Training time is for the real-only training set (1427 participants); training of the neural models is reported in Table 15 and in the supplementary results.

**Table 31:** Federated communication volume and per-round overhead (identical definitions and units to Table 32).

System	Rounds	Update (KiB)	Uplink/ Round (KiB)	Downlink/ Round (KiB)	Total, Both Directions (MiB)	Parameters	Extra Overhead (ms/Round)
FedAvg	30	38.50	500.55	500.55	29.329	9857	Not applicable
FedProx	30	38.50	500.55	500.55	29.329	9857	Not applicable
HIV-PrivFed (DP-FedProx)	30	38.50	500.55	500.55	29.329	9857	33.9 (optional masking and AES-GCM, Table 32)

All systems use the same 9857-parameter ResMLP-DP, 30 rounds, and the same 13 clients in every round. Update size: 9857 float32 values =39,428 bytes. Per round, the uplink carries 13 updates and the downlink 13 copies of the global model, both counted in plaintext (the optional authentication tag and nonce add 32 bytes per message). Units: 1 KiB =1024 bytes, 1 MiB $=1024^{2}$ bytes. Total =60×13×39,428 bytes, uplink and downlink. The extra overhead is the measured computation time of the optional protections; differential privacy itself adds no communication.

**Table 32:** Pairwise-masking secure aggregation and AES-GCM overhead (simulation) measured for 13 clients.

Metric	Value
Update size, plaintext (bytes)	39,428
Update size, plaintext (KiB)	38.50
Masked update size (bytes, same dimension)	39,428
Ciphertext incl. tag and nonce (bytes)	39,460
Masking, all 13 clients (ms), mean/P95	28.56/51.69
Server aggregation (ms), mean/P95	0.206/0.355
AES-GCM encryption per client (ms), mean/P95	0.241/0.310
AES-GCM decryption per client (ms), mean/P95	0.157/0.307
Total per-round overhead (ms): masking + aggregation + 13× encryption + 13× decryption, mean/P95	33.95/61.21
Uplink per round, 13 clients (KiB)	500.55
Uplink over 30 rounds (MiB)	14.665
Aggregation correctness (maximum absolute error versus the unmasked mean)	3.58 × 10^−7^

Measured on one virtual CPU core over 100 repetitions. This is a single-process simulation (shared-seed pairwise masks, no key agreement, no dropout handling, no authentication) and measures computational overhead only; it is not a deployable secure aggregation protocol. Units: 1 KiB =1024 bytes, 1 MiB $=1024^{2}$ bytes.

**Table 33:** Testing prioritization utility, showing positives found when only the highest-risk participants are tested.

Model	Top 10%	Top 20%	Top 30%	Top 40%	Top 50%
EasyEnsemble (real-only)	26% (27.1%)	42% (22.2%)	54% (19.1%)	63% (16.5%)	73% (15.4%)
Logistic regression (real-only)	26% (27.1%)	41% (21.4%)	57% (19.9%)	66% (17.4%)	76% (16.0%)
FedProx (non-private)	18% (18.9%)	32% (16.9%)	44% (15.4%)	53% (14.0%)	63% (13.2%)
FedAvg (non-private)	18% (18.6%)	32% (16.9%)	44% (15.3%)	53% (14.0%)	63% (13.2%)
HIV-PrivFed sigma=0.8 (tuned lr)	19% (20.5%)	35% (18.4%)	48% (16.9%)	59% (15.7%)	70% (14.8%)

Each cell: share of all HIV-positive test participants found among the top-ranked fraction (positive predictive value in parentheses). Test prevalence 10.6%, so random ordering finds the same share as the fraction tested with PPV 10.6%. Neural rows: mean over five seeds.

## Data Availability

The Sialon-II dataset analyzed in this study is available from [3] subject to the access conditions of the original study. Code and analysis scripts implementing the preprocessing, modeling, federated training, differential privacy, and secure aggregation pipelines described in this manuscript are available at https://github.com/mdwahidur-rahman/HIV-PrivFed-Federated-Learning-for-HIV-Risk-Stratification or from the corresponding author upon reasonable request. The repository version corresponding to this manuscript is tagged (release v2.0), and a SHA-256 manifest of all code and result files is provided together with the scripts and per-run results of the revised experiments in the Supplementary Materials.

## Supplementary Materials

The following supporting information can be downloaded at: https://www.mdpi.com/article/10.3390/diagnostics16213590/s1.

## Abbreviations

The following abbreviations are used in this manuscript:

AIDS	Acquired Immunodeficiency Syndrome
AES-GCM	Advanced Encryption Standard–Galois/Counter Mode
AUPRC	Area Under the Precision–Recall Curve
AUROC	Area Under the Receiver Operating Characteristic Curve
BCE	Binary Cross-Entropy
BRF	Balanced Random Forest
C2ST	Classifier Two-Sample Test
CI	Confidence Interval
DP	Differential Privacy
DP-SGD	Differentially Private Stochastic Gradient Descent
ECE	Expected Calibration Error
FL	Federated Learning
FedAvg	Federated Averaging
FedProx	Federated Proximal Optimization
FPR	False Positive Rate
GN	Group Normalization
HIV	Human Immunodeficiency Virus
JSD	Jensen–Shannon Distance
LR	Logistic Regression
MCC	Matthews Correlation Coefficient
MIA	Membership Inference Attack
ML	Machine Learning
MLP	Multi-Layer Perceptron
MMD	Maximum Mean Discrepancy
MSM	Men Who Have Sex with Men
non-IID	Non-Identically and Independently Distributed
PRV	Privacy Loss Random Variable
RDP	Rényi Differential Privacy
ReLU	Rectified Linear Unit
ResMLP	Residual Multilayer Perceptron
RF	Random Forest
RBF-SVM	Radial Basis Function Support Vector Machine
SMD	Standardized Mean Difference
SMOTENC	Synthetic Minority Oversampling Technique for Nominal and Continuous Features
STI	Sexually Transmitted Infection
t-SNE	t-Distributed Stochastic Neighbor Embedding
TPR	True Positive Rate
WHO	World Health Organization
